# The potential clinical utility of Whole Genome Sequencing for patients with cancer: evaluation of a regional implementation of the 100,000 Genomes Project

**DOI:** 10.1038/s41416-024-02890-6

**Published:** 2024-10-30

**Authors:** Elaine Y. L. Leung, Helen L. Robbins, Shafquat Zaman, Neeraj Lal, Dion Morton, Lisa Dew, Anthony P. Williams, Yvonne Wallis, Jennie Bell, Manoj Raghavan, Gary Middleton, Andrew D. Beggs

**Affiliations:** 1 Institute of Cancer and Genomic Sciences, University of Birmingham, Birmingham, UK; 2 Institute of Immunology and Immunotherapy, University of Birmingham, Birmingham, UK; 3 Institute of Applied Health Research, University of Birmingham, Birmingham, UK; 4 Central and South Genomic Medicine Service Alliance, Birmingham, UK; 5 The Wessex NHS Genomics Medicine Centre (WGMC), the University of Southampton, Southampton, UK; 6 The West Midlands Regional Genomics Laboratory (WMRGL), Birmingham Women’s and Children’s NHS Foundation Trust, Birmingham, UK

**Keywords:** Molecular medicine, Genetics, Cancer genetics, Oncology

## Abstract

**Background:**

The 100,000 Genomes Project established infrastructure for Whole Genome Sequencing (WGS) in the United Kingdom.

**Methods:**

A retrospective study of cancer patients recruited to the 100,000 Genomes Project by the West Midlands Genomics Medicine Centre, evaluating clinical relevance of results.

**Results:**

After excluding samples with no sequencing data (1678/4851; 34.6%), 3166 sample sets (germline and somatic) from 3067 participants were sequenced. Results of 1256 participants (41.0%) were interpreted (excluding participants who died (308/3067; 10.0%) or were clinically excluded (1503/3067; 49.0%)). Of these, 323 (25.7%) had no variants in genes which may alter management (Domain 1 genes). Of the remaining 933 participants, 552 (59.2%) had clinical recommendations made (718 recommendations in total). These included therapeutic recommendations (377/933; 40.4%), such as clinical trial, unlicensed or licensed therapies or high TMB recommendations, and germline variants warranting clinical genetics review (85/933; 9.1%). At the last follow up, 20.2% of all recommendations were followed (145/718). However, only a small proportion of therapeutic recommendations were followed (5.1%, 25/491).

**Conclusions:**

The 100,000 Genomes Project has established infrastructure and regional experience to support personalised cancer care. The majority of those with successful sequencing had actionable variants. Ensuring GTAB recommendations are followed will maximise benefits for patients.

## Background

Cancers are complex genetic diseases caused by dysregulation of multiple molecular pathways [1]. Comprehensive molecular profiling is as an essential component of personalised cancer care delivery. Internationally, most genomics services use a combination of different sequencing techniques according to tumour type and standard of care pathways. In the United Kingdom, the National Health Service of England (NHSE), through its subsidiary Genomics England, aimed to provide whole-genome sequencing (WGS): a single assay that provides the most genetic information, within a clinically relevant timeframe. The recently completed 100,000 Genomes Project [2]. established histopathological (tissue acquisition and processing) and genomic (data generation, analysis and interpretation) infrastructures to enable WGS to be incorporated into routine clinical practice within the UK National Health Service.

WGS has demonstrated its remarkable success in the diagnosis of rare diseases [2, 3]. In addition, WGS has demonstrated clinical benefit in the management of children with cancers [4] and has enhanced scientific understanding of mutational signatures in cancers [5]. WGS offers the opportunity for a data-rich singular assay for adult cancer, however, the clinical utility of WGS in adult cancer is poorly described. The detailed mutational characteristics of successfully sequenced specimens during the 100,000 Genomes Project have recently been described [6], however, relevant questions remain underexplored including timing of testing (in relation to standard of care treatments) and whether reported results can be integrated into clinical practice and potential treatments. For example, it is important to evaluate the balance between turnaround time of WGS and the time to deterioration in a heavily pretreated patients, as well as the disparity in availability of clinical trial and unlicensed therapies [7, 8].

The West Midlands Genomic Medicine Service Alliance covers a geographically wide and diverse population encompassing the country’s first “majority minority” city in Birmingham, and includes extremes of socioeconomic status. This population is therefore incredibly meaningful when assessing the clinical applicability of WGS. This study aimed to evaluate the experience of participants with cancers who were enroled in the 100,000 Genomes Project via the West Midlands Genomics Medicine Centre (GMC). Crucially, we analysed the discussion outcomes of the multidisciplinary Genomic Tumour Advisory Boards (GTABs), exploring the potential clinical utility of WGS in cancer care.

## Materials and methods

Study design and overview of the 100,000 Genomes Project. This is a retrospective evaluation of data collated during the 100,000 Genomes Project (‘the Project’), a national initiative [7], which prospectively recruited patients with rare disease or cancer between 2012 and 2018. The primary objective of the Project was to sequence 100,000 whole genomes from patients with cancer and rare disease, establishing infrastructure to embed genomics into the National Health Service (NHS). More widely, the Project sought to accelerate the growth and understanding of genomic medicine, bring clinical benefits through improving diagnosis and treatment, and support scientific discovery.

During the Project, thirteen NHS Genomic Medicine Centres (GMCs) were set up across the country with linked GTABs for multidisciplinary team (MDT) discussions and recommendations of management. After approval from the national research ethics committee, participants were identified by healthcare professionals and researchers. To date, more than 100,000 genomes have been sequenced from over 97,000 participants. This study aimed to evaluate the clinical utility of WGS for patients with cancers recruited to the Project in the West Midlands by assessing the variants identified by WGS and subsequent clinical recommendations.

### West Midlands Genomic Medicine Centre

While the Project was launched in 2012 and national recruitment commenced in 2014, the West Midlands Genomic Medicine Centre (WMGMC) was established in December 2014. Recruitment within the WMGMC ran between 2015 and 2018, with all recruitment undertaken by dedicated research staff, as reported previously [2]. Routine patient care was unchanged. Parallel recruitment to the Project and other clinical trials was allowed.

### Inclusion and exclusion criteria

Individuals eligible for the 100,000 Genomes Project were those diagnosed with invasive malignancy, residing in one of the 4 nations of the United Kingdom (England, Scotland Northern Ireland or Wales) and receiving cancer care within NHS England. Eligibility was not determined by tumour stage, tumour grade, or expected prognosis. Patients unable to consent were excluded. Additional exclusion criteria included: previously receiving negative results from another WGS study; unavailability of the essential dataset, known familial cancer syndromes. Ineligible cancers included: cervical, vaginal and vulval cancers (except melanoma), cancers of the endocrine system (except thyroid cancers), squamous cell or basal cell skin cancers, placental malignancies, cardiac malignancy and cancers from the male genital tract (except prostate, testicular or melanoma). Individuals with haematological malignancy were eligible, broadly including patients with haematological malignancy with >40% malignant nuclei on peripheral blood or bone marrow for whom imminent treatment was planned. Full inclusion and exclusion criteria for the 100,000 Genomes Project are available from: https://www.genomicsengland.co.uk/initiatives/100000-genomes-project/documentation, and are outlined in Supplementary Methods (Supplementary Materials).

### Laboratory procedures and analysis

Details of procedures, quality control and analysis are described in Supplementary Methods (Supplementary Materials), and have been reported elsewhere [9]. Samples were handled according to the Project’s standard operating procedures by the West Midlands Regional Genetic Laboratory (WMRGL). Sequencing and primary analysis were performed by Illumina (Cambridge, United Kingdom), reporting triaged variant calls to the regional GMC. Variants were reported according to virtual panels of genes in 3 different domains (Supplementary Table 1–7; Supplementary Methods (Supplementary Materials) and https://panelapp.genomicsengland.co.uk/), which are driven by expert consensus [10]. Domain 1 included a list of clinically actionable genes. Actionable genes are defined by the GenomOncology Knowledge Management System as genes that have reported therapeutic, prognostic or clinical trial associations. Domain 2 variants were non-actionable cancer-related genes that have been causally implicated in cancer by the Cancer Gene Census (http://cancer.sanger.ac.uk/census). Other variants not included in Domains 1 and 2 are reported as Domain 3 variants (non-cancer gene variants). Variants in Domain 1 gene were reviewed at GTAB for potential significance.

### West Midlands Genomic Tumour Advisory Boards (GTABs)

GTABs were convened across three groupings of tumour sites. GTAB1 consisted of urological, skin, breast, head & neck and lung cancers, GTAB2 consisted of haematological, brain & paediatric cancers, sarcoma and carcinoma of unknown primary (CUP). GTAB3 consisted of colorectal, gynaecological, upper GI, neuroendrocrine and hepatobiliary cancers. All GTABs were chaired by a consultant medical or surgical oncologist, and consisted of a consultant histopathologist, clinical scientist, oncologist, surgeon and administrator. GTABs met bi-weekly.

Pre-GTAB filtering sought to identify participants who would not benefit from GTAB review (deceased participants with no germline variants, alive participants without somatic domain 1 variants, participants on treatment with ongoing response, participants who have completed active cancer management). The primary role of GTAB was to triage results of WGS, reviewing results of participants who were alive, with at least one somatic domain 1 variant and who were undergoing ongoing cancer management. All pertinent germline variants were reviewed at GTAB (including for deceased participants). Potential clinical actionability was defined at GTAB as variants in Domain 1 genes (as defined by Genomics England PanelApp) and classified by ClinVar as likely pathogenic, according to American College of Medical Genetics (ACMG) recommendations [11, 12]. Potentially clinically actionable findings were validated by orthogonal testing, before being reported back to subsequent meetings. Potential eligibility for stratified medicine trials were identified through Genomics England PanelApp linked with ClinicalTrials.gov. A schema for interpretation of WGS results through pre-GTAB filtering and GTAB is provided in Supplementary Fig. 1. Recommendations from GTAB were sent to local treating clinicians, with follow-up of recommendations requested from treating clinicians to assess whether recommendations altered clinical management.

### Study outcomes

The primary outcome evaluated in this study was the proportion of participants with clinically actionable outcomes and their subsequent management. The secondary outcomes include the proportions and causes of the lack of successful genomic test results. Outcomes were further analysed according to the year of recruitment and cancer type.

### Data collection, definitions and follow up

The data were collated from the relevant GTABs and the WMRGL. Participants were defined as clinically excluded if poor performance status precluded additional therapies, or if disease status or stage (e.g., early stage, non-metastatic, curatively treated, not on active cancer treatment) meant that sequencing data was unlikely to change their care. The last follow-up of participant outcomes was completed on 31 December 2021.

### Statistical analysis

Collated data were checked for consistency, cleaned and exported for analysis. The study was conducted according to guidelines set by the STROBE (Strengthening the Reporting of Observational Studies in Epidemiology) statement for observational studies [13]. Chi-square tests were used to compare differences in categorical data. Missing data are included in summary tables when applicable. Analysis and visualisation were performed used Stata SE version 16.1 (StataCorp, Texas, USA) and GraphPad Prism 9 (Insight Partners, New York, USA).

## Results

### Participants and tumour-related characteristics

**Fig. 1:**
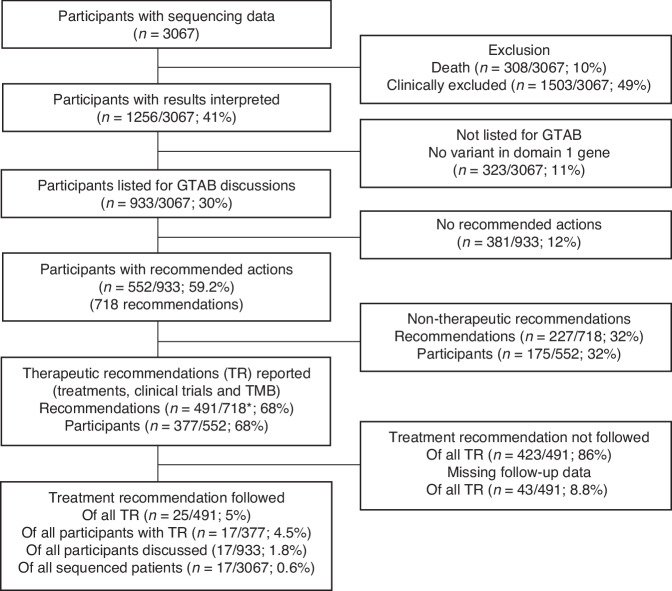
A flow chart summarising the number of participants and the outcomes after therapeutic recommendations. *Multiple recommendations per participants were made for a proportion of this cohort. TR= Therapeutic recommendations (clinical trial, unlicensed or licensed therapies or high TMB).

4851 sample sets (comprising a somatic and paired germline sample) from 4830 participants, representing 14 cancer types (Fig. 1, Supplementary Fig. 2 and Supplementary Table 8) were included in this analysis. Over half of samples were collected in the final year of the study (2767/4851; 57.0%; Supplementary Fig. 3). The commonest cancer types were colorectal cancer (977/4842; 20.2%), breast cancer (933/4842; 19.3%) and urological cancers (680/4842; 14.0%), accounting for over half of total samples (2590/4842; 53.5%; Table 1 and Supplementary Table 8). Minimum age was 1.8 years, maximum was 97.8 years, with a median of 66.3 years. 50.1% were male (2465/4851). Median PS was 1 (range 0– 4). Disease stage was not routinely recorded.

**Table 1:** Summary outcomes of participants with samples sent by cancer types (*n* = 3067)

	All (*n* = 3067)	Colorectal (*n* = 711)	Breast (*n* = 603)	Urology (*n* = 439)	Sarcoma (*n* = 308)	HPB (*n* = 266)	Lung (*n* = 201)	Glioma (*n* = 127)	Blood (*n* = 79)	Melanoma (*n* = 106)	Upper GI (*n* = 96)	H&N (*n* = 33)	Gynae (*n* = 47)	Paediatric (*n* = 34)	CUP (*n* = 17)	*p* value
Samples sequenced, without result interpreted at last follow up																
Death	308 (17.0)	53 (11.0)	15 (3.5)	40 (16.1)	30 (25.4)	36 (31.3)	40 (28.0)	32 (57.1)	9 (18.4)	22 (44.0)	17 (28.8)	5 (23.8)	4 (18.2)	0(0)	5^50^	<0.0001
Clinically Excluded	1503 (83.0)	427 (89.0)	410 (96.5)	208 (83.9)	88 (74.6)	79 (68.7)	103^72^	24 (42.9)	40 (81.6)	28 (56.0)	42 (71.2)	16 (76.2)	18 (81.8)	15 (100.0)	5^50^	<0.0001
**Total**^**a**^	**1811 (59.1)**	**480 (67.5)**	**425 (70.5)**	**248 (56.5)**	**118 (38.3)**	**115 (43.2)**	**143 (71.1)**	**56 (44.1)**	**49 (26.5)**	**50 (47.2)**	**59 (61.5)**	**21 (63.6)**	**22 (30.1)**	**15 (44.1)**	**10 (58.8)**	<0.0001
Samples sequenced, with results interpreted at the last follow up																
No actionable Domain 1 variant	323 (25.7)	30 (13.0)	71 (39.9)	94 (49.2)	24 (12.6)	49 (18.4)	17 (29.3)	3 (4.2)	0(0)	8 (14.3)	12 (32.4)	6 (50.0)	2 (10.5)	2 (10.5)	5 (71.4)	<0.0001
Results interpreted	933 (74.3)	201 (87.0)	107 (60.1)	97 (50.8)	166 (87.4)	102 (81.6)	41 (70.7)	68 (95.8)	30 (100)	48 (85.7)	25 (67.6)	6 (50.0)	23 (89.5)	17 (89.5)	2 (28.6)	<0.0001
**Total**^**a**^	**1256 (40.9)**	**231 (32.5)**	**178 (29.5)**	**191 (43.5)**	**190 (61.5)**	**151 (56.8)**	**58 (28.9)**	**71 (55.9)**	**30 (38.0)**	**56 (52.8)**	**37 (38.5)**	**12 (36.4)**	**25 (53.2)**	**19 (55.9)**	**7 (41.2)**	<0.0001

Count (percentage of category).

*HPB* hepatobiliary, *Blood* haematological, *GI* gastrointestinal tract, *H&N* head and neck, *Gynae* Gynaecological, *CUP* Cancer of Unknown Primary.

^a^Count (Percentage of column total).

### Proportion of sample sets sent for WGS

**Fig. 2:**
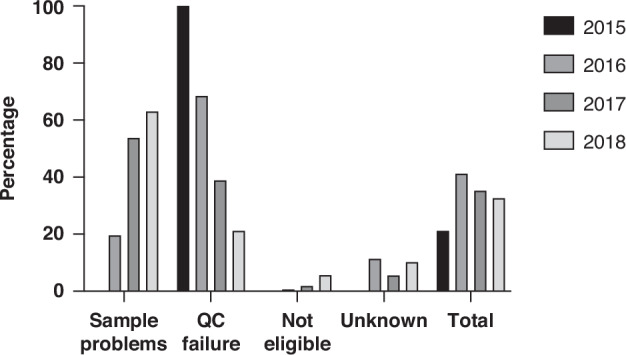
A summary of stated reasons when samples were not sent for sequencing over time (*n* = 1669)*.

3166 sample sets (65.3%) from 3067 participants were sent for sequencing (Fig. 1, Table 1, Supplementary Table 8). The majority of the 1669 samples were not sent for sequencing were due to sample issues (913/1669; 54.7%): 510/1669 samples were insufficient due to low tumour cellularity in the specimen, with the remainder (403/1669) having insufficient mass for DNA extraction. A third of the samples that were not sent for sequencing failed quality control (538/1669; 32.2%), this rate significantly reduced over time (Fig. 2, *p* < 0.0001). Median turnaround time for WGS was 16 weeks (range 4–18 weeks), reducing to 4 weeks by the end of the study period.

### Clinical exclusions and Pre-GTAB Filtering

Approximately 6 in 10 (59.1%; 1811/3067) participants with sequencing results were not discussed at GTAB because results were unlikely to change outcomes (Table 1). 10% of participants with sequencing results died before interpreted results were available (308/3067), including: 29.4% of participants with Cancer of Unknown Primary (CUP; 5/17); 25.2% of participants with adult glioma (32/127); 20.8% of melanoma (22/106); 19.9% of lung cancer (40/201) and 17.7% of upper gastrointestinal tract cancers (17/96). A further 50% of participants (1503/3067) were not discussed at GTAB as discussion was unlikely to change clinical outcome (for example not being on active treatment for cancer, not having active cancer, or due to poor performance status). After filtering out of participants who were clinically excluded, 1256 participants were eligible for GTAB discussion. Of the 1256 participants clinically eligible for GTAB 25.7% (323/1256) were not discussed due to absence of variants in potentially actionable (domain 1) genes.

### GTAB recommendations

993 participants had sequencing results were discussed (Table 2), of whom 55.5% (552/993) had a recommendation made. In total, 718 recommendations were made. The majority of GTAB recommendations were related to potential treatment (both licensed and unlicensed) options and clinical trials. Only likely pathogenic variants in domain 1 genes were considered when recommend potential therapies. In total, 40.4% (377/933) of participants discussed had a recommendation that could relate to a potential treatment option (32.3% (301/933) after exclusion of high TMB reports). Of participants who had a recommendation made, 4.3% (24/522) had a recommended licensed therapy, 3.4% (19/522) had a recommended unlicensed therapy, 49.6% (274/552) had a recommended clinical trial, and 17.4% (96/522) had a report of high TMB. Gene variants directing therapeutic options (clinical trials, licensed or unlicensed therapies) are summarised in Supplementary Fig. 4.

**Table 2:** A summary of all recommendations of cases with reported results according to cancer type (*n* = 933 and 718 recommendations)

	All (*n* = 993)	Colorectal (*n* = 201)	Breast (*n* = 107)	Urology (*n* = 97)	Sarcoma (*n* = 166)	HPB (*n* = 102)	Lung (*n* = 41)	Glioma (*n* = 68)	Blood (*n* = 30)	Melanoma (*n* = 48)	Upper GI (*n* = 25)	H&N (*n* = 6)	Gynae (*n* = 23)	Paediatric (*n* = 17)	CUP (*n* = 2)	*p* value
Participants with reported results																
No recommendation	381 (38.4)	71 (35.3)	25 (23.4)	43 (44.3)	108 (65.1)	36 (35.3)	8 (19.5)	24 (35.3)	18 (60.0)	18 (37.5)	12 (48.0)	2 (33.3)	9 (39.1)	6 (35.3)	1 (50.0)	<0.0001
Recommendation	552 (61.6)	130 (64.7)	82 (76.6)	54 (55.6)	58 (34.9)	66 (64.7)	33 (80.5)	44 (64.7)	12 (40.0)	30 (62.5)	13 (52.0)	4 (66.7)	14 (60.9)	11 (64.7)	1 (50.0)	<0.0001
Participants with therapeutic recommendations^b^																
Treatment (Licensed)	24 (4.3)	3 (2.3)	4 (4.9)	4 (7.4)	0(0)	2 (3.0)	2 (6.1)	1 (2.3)	0(0)	5 (16.7)	1 (7.7)	0(0)	2 (14.3)	0(0)	0(0)	0.0234
Treatment (Unlicensed)	19 (3.4)	1 (0.8)	6 (7.3)	4 (7.4)	0(0)	0(0)	1 (3.0	0 (0.0)	0(0)	5 (16.7)	0(0)	1 (25.0)	0(0)	1 (9.1)	0(0)	<0.0001
Clinical Trial	274 (49.6)	60 (46.2)	34 (41.5)	21 (38.9)	26 (44.8)	46 (69.7)	22 (66.7)	31 (70.5)	4 (33.3)	15 (50.0)	5 (38.5)	1 (25.0)	8 (57.1)	0(0)	0(0)	<0.0001
High TMB report	96 (17.4)	48 (36.9)	3 (3.7)	11 (20.4)	0(0)	4 (6.1)	6 (18.2)	3 (6.8)	0(0)	13 (43.3)	3 (23.1)	0(0)	4 (28.6)	0(0)	1 (100.0)	<0.0001
Participants with other recommendations																
DPYD	56 (10.1)	8 (6.2)	11 (13.4)	7 (13.0)	9 (15.5)	0(0)	8 (24.2)	0(0)	6 (50.0)	2 (6.7)	1 (7.7)	1 (25.0)	1 (7.1)	2 (18.2)	0(0)	<0.0001
Clinical genetics	85 (15.4)	20 (15.4)	27 (32.9)	9 (16.7)	11 (19.0)	7 (10.6)	1 (3.0)	3 (6.8)	2 (16.7)	1 (3.3)	0(0)	0(0)	0(0)	4 (36.4)	0(0)	<0.0001
Not validated	86 (15.6)	6 (4.6)	14 (17.1)	8 (14.8)	18 (31.0)	11 (16.7)	5 (15.2)	9 (20.5)	2 (16.7)	3 (10.0)	4 (30.8)	2 (50.0)	0(0)	4 (36.4)	0(0)	0.0110
*Participants with therapeutic recommendations*^c^	377/552 (68.3)	110/130 (84.6)	43/82 (52.4)	33/54 (61.1)	26/58 (44.8)	50/66 (75.8)	25/33 (75.8)	34/44 (77.2)	4/12 (33.3)	27/48 (56.3)	9/13 (69.2)	2/4 (50.0)	12/14 (85.7)	1/11 (9.1)	1/1 (100.0)	<0.0001
Therapeutic recommendations^b^																
Treatment (Licensed)	26 (3.6)	3 (1.8)	5 (4.6)	4 (5.6)	0(0)	2 (2.5)	2 (3.6)	1 (1.7)	0(0)	6 (12.2)	1 (6.7)	0(0)	2 (12.5)	0(0)	0(0)	0.059
Treatment (Unlicensed)	21 (2.9)	1 (0.6)	6 (5.5)	4 (5.6)	0(0)	0(0)	1 (1.8)	0(0)	0(0)	7 (14.3)	0(0)	1 (20.0)	0(0)	1 (9.1)	0(0)	<0.0001
Clinical Trial	348 (48.5)	77 (47.2)	43 (39.4)	28 (39.4)	30 (44.1)	56 (70.0)	33 (58.9)	44 (73.3)	4 (28.6)	17 (34.7)	6 (40.0)	1 (20.0)	9 (56.3)	0(0)	0(0)	<0.0001
TMB report	96 (13.4)	48 (29.4)	3 (2.8)	11 (15.5)	0(0)	4 (5.0)	6 (10.7)	3 (5.0)	0(0)	13 (26.5)	3 (20.0)	0(0)	4 (25.0)	0(0)	1 (100.0)	<0.0001
Other recommendations																
DPYD	56 (7.8)	8 (4.9)	11 (10.1)	7 (9.9)	9 (13.2)	0(0)	8 (14.3)	0(0)	6 (42.9)	2 (4.1)	1 (6.7)	1 (20.0)	1 (6.3)	2 (18.2)	0(0)	<0.0001
Clinical genetics	85 (11.8)	20 (13.3)	27 (24.8)	9 (12.7)	11 (16.2)	7 (8.8)	1 (1.8)	3 (5.0)	2 (14.3)	1 (2.0)	0(0)	0(0)	0(0)	4 (36.4)	0(0)	<0.0001
Not validated	86 (12.0)	6 (3.7)	14 (12.8)	8 (11.3)	18 (26.5)	11 (13.8)	5 (8.9)	9 (15.0)	2 (14.3)	3 (6.10)	4 (26.7)	2 (40.0)	0(0)	4 (36.4)	0(0)	<0.0001
*Total number of all recommendations*^c^	718	163	109	71	68	80	56	60	14	49	15	5	16	11	1	<0.0001
*Total number of all therapeutic recommendations*	491/718 (68.4)	129/163 (79.1)	57/109 (52.2)	47/71 (66.2)	30/68 (44.1)	62/80 (77.5)	42/56 (75.0)	48/60 (80.0)	4/14 (28.6)	43/49 (87.8)	10/15 (33.3)	2/5 (40.0)	15/16 (93.8)	1/11 (9.1)	1/1 (100.0)	<0.0001

Count (Percentage of total recommended action).

*HPB* hepatobiliary, *Blood* haematological, *GI* gastrointestinal tract, *H&N* head and neck, *Gynae* Gynaecological, *CUP* Cancer of Unknown Primary, *DPYD* dihydropyrimidine dehydrogenase polymorphism, *TMB* Tumour mutational burden.

^a^Count (Proportion of total indicated).

^b^therapeutic recommentations= trial, licensed or unlicensed treatment, high TMB.

^c^Multiple recommendations per participants were made for a proportion of this cohort.

Different cancer types were associated with differing rates of recommended actions (Table 2). Of participants discussed at GTAB: 53.7% (22/41) of lung cancer participants, 46.1% (47/102) of hepato-pancreatico-biliary cancer participants and 47.1% (32/68) of adult glioma participants had potential treatment or trial recommendations. In comparison, only 15.7% (26/166) of sarcoma participants and 13.3% (4/30) of haemato-oncology participants discussed at GTAB had potential treatment or trial recommendations.

Supplementary Table 9 outlines recommended licensed and unlicensed therapy options from GTAB (*n* = 144) by genetic aberrations. Checkpoint blockade was the most commonly recommended therapy (97/144), overwhelmingly recommended for high TMB (96/97). PI3K/Akt/mTOR inhibitors was recommended in 14.6% (21/144) most commonly due to mutations in *PIK3CA* (*n* = 10), *MTOR* (*n* = 5), or *TSC1* (*n* = 2). *BRAF* and/or *MEK* inhibition was recommended in 8.3% (12/144), most commonly due to *BRAF* mutation. Consideration of PARP inhibition was recommended in 5.5% (8/144). *KRAS* inhibitors were recommended in two cases (1.4%).

Participants with colorectal cancer (48/200; 24.0%) were more likely to have a high TMB, compared to an overall rate of 10.3% (96/928), consistent with the expected number of dMMR and *POLE/D1* mutant participants. A high TMB may predict response to immunotherapy, although TMB remains an imperfect biomarker [14–16]. Of note, although a significant proportion of participants with melanoma discussed at GTAB: (13/48; 27.1%) had high TMB, although this is lower than reported elsewhere (~50%) [17, 18].

Participants with breast and paediatric cancers were more likely to be referred to clinical genetic services. Referral to clinical genetics was recommended for a quarter of participants with childhood cancers discussed at GTAB (4/16, 25.0%) and quarter of breast cancer participants discussed at GTAB (27/106, 25.5%). Supplementary Table 10 summarises germline variants triggering recommendations for clinical genetics referral (*n* = 82), the most common being *BRCA2* (21/82), *MSH2* (11/82), *ATM* (8/82), *BRIP1* (7/82), *PALB2* (5/82), *TP53* (4/82), and *BRCA1* (4/82).

348 clinical trials recommendations were made in 274 participants (Supplementary Table 11). Trials recommended by GTAB were predominantly Phase I or II trials of targeted therapy options. Reflecting the frequency of *TP53* mutations, the most commonly recommended trial was NCT03096054, a Phase I trial of LY3143921, a CDC7 inhibitor (76/348, 21.8%) [19].

### Outcomes for Clinical Trial Recommendations

GTAB recommendations were sent to treating clinicians between September 2018 and February 2021, with 99.5% (715/718) of recommendations sent before the end of October 2020. Treating clinicians were asked to provide feedback on whether recommendations were followed, with follow-up completed in December 2021.

**Fig. 3:**
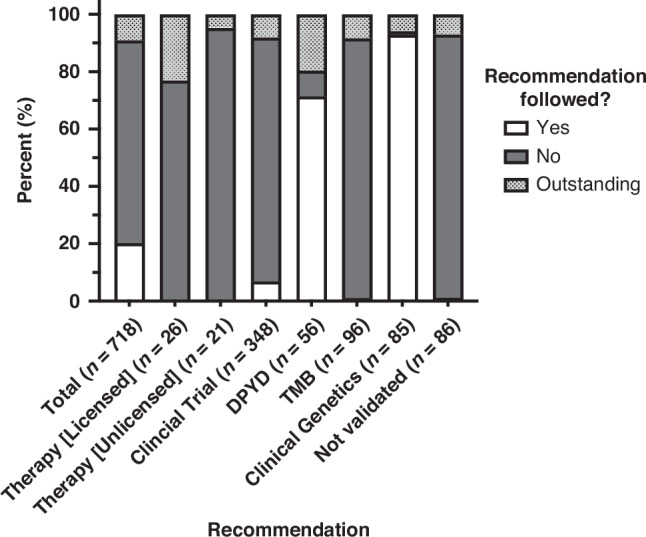
Proportions of recommendations followed, based on the type of recommendation (*n* = 718).

Figure 3, Table 3 and Supplementary Table 12 summarise outcomes of recommendations. Feedback was complete for 90.9% (653/718) of recommendations. 22.2% (145/653) of all recommendations were followed. 98.8% (79/80) of recommendations to refer to clinical genetics were followed, 88.9% (40/45) of participants with DPYD deficiency were informed of the result. However, rates of following recommendations for therapy options were lower: only 5.6% (25/448) of therapeutic recommendations (licensed or unlicensed therapies, clinical trials, high TMB) were followed. 7.5% of recommendations to refer for a clinical trial were followed (24/320). Therapeutic recommendations were followed for 17 patients (representing 25 recommendations, as some patients had multiple clinical trials recommended). 16 participants were referred clinical trials (4 patients with sarcoma, 3 with colorectal cancer, 2 with glioma, 2 with breast cancer, 2 with HPB cancers, 1 with gynaecological malignancies, 1 with lung cancer, 1 with UGI malignancy). One participant with *ERBB2*-mutant breast cancer who was recommended for clinical trials was started on treatment with neratinib on compassionate basis - this was overall judged to be consistent with following the two GTAB recommendations to consider clinical trials for access to *ERRB2*-directed therapy for this participant.

**Table 3:** Summary of clinical follow up of therapeutic recommendations (trial, licensed or unlicensed treatment, high TMB) by cancer type (n = 491)

	All (*n* = 491)	Colorectal (*n* = 129)	Breast (*n* = 57)	Urology (*n* = 47)	Sarcoma (*n* = 30)	HPB (*n* = 62)	Lung (*n* = 42)	Glioma (*n* = 48)	Blood (*n* = 4)	Melanoma (*n* = 43)	Upper GI (*n* = 10)	H&N (*n* = 2)	Gynae (*n* = 15)	Paediatric (*n* = 1)	CUP (*n* = 1)	*p* value
Therapeutic recommendations followed																
Referred to Trial	23 (92.0)	5 (100.0)	3 (60.0)	0	6 (100.0)	2 (100.0)	1 (100.0)	3 (100.0)	0(0)	0(0)	1 (100.0)	0(0)	2 (100.0)	0(0)	0(0)	<0.05
Compassionate Treatment	2 (8.0)	0(0)	2 (40.0)	0	0(0)	0(0)	0(0)	0(0)	0(0)	0(0)	0(0)	0(0)	0(0)	0(0)	0(0)	ns
**Total**^**a**^	**25 (5.1)**	**5 (3.9)**	**5 (8.8)**	**0**	**6 (20.0)**	**2 (3.2)**	**1 (2.4)**	**3 (6.3)**	**0(0)**	**0(0)**	**1 (10.0)**	**0(0)**	**2 (13.3)**	**0(0)**	**0(0)**	**<0.05**
Therapeutic recommendations not followed																
Death	72 (17.0)	18 (15.5)	3 (6.4)	6 (16.2)	0(0)	16 (27.1)	15 (44.1)	8 (17.8)	0(0)	4 (11.4)	2 (22.2)	0(0)	0(0)	0(0)	0(0)	<0.001
Participant not fit / suitable	38 (9.0)	12 (10.3)	0(0)	5 (13.5)	2 (8.7)	6 (10.2)	1 (2.9)	5 (11.1)	0(0)	4 (11.4)	2 (22.2)	0(0)	1 (9.1)	0(0)	0(0)	ns
No disease	173 (40.9)	56 (48.3)	23 (48.9)	14 (37.8)	16 (69.6)	20 (33.9)	10 (29.4)	12 (26.7)	0(0)	11 (31.4)	2 (22.2)	1 (50.0)	7 (63.6)	0(0)	1 (100)	<0.05
No change to treatment	136 (32.2)	29 (25.0)	21 (44.7)	12 (32.4)	5 (21.7)	15 (25.4)	8 (23.5)	20 (44.4)	3 (100.0)	16 (45.7)	3 (33.3)	1 (50.0)	2 (18.2)	1 (100.0)	0(0)	<0.05
Participant declined	4 (0.9)	1 (0.9)	0(0)	0(0)	0(0)	2 (3.4)	0(0)	0(0)	0(0)	0(0)	0(0)	0(0)	1 (9.1)	0(0)	0(0)	<0.05
**Total**^**a**^	**423 (86.2)**	**116 (89.9)**	**47 (82.5)**	**37 (78.7)**	**23 (76.7)**	**59 (95.2)**	**34 (81.0)**	**45 (93.8)**	**3 (75.0)**	**35 (81.4)**	**9 (90.0)**	**2 (100.0)**	**11 (73.3)**	**1 (100.0)**	**1 (100)**	**ns**
Incomplete data^a^	**43 (8.8)**	**8 (6.2)**	**5 (8.8)**	**10 (21.3)**	**1 (3.3)**	**1 (1.6)**	**7 (16.7)**	**0(0)**	**1 (25.0)**	**8 (18.6)**	**0(0)**	**0(0)**	**2 (13.3)**	**0(0)**	**0(0)**	< **0.005**

Count (percentage of category).

*HPB* hepatobiliary, *Blood* haematological, *GI* gastrointestinal tract, *H&N* head and neck, *Gynae* Gynaecological, *CUP* Cancer of Unknown Primary, *DPYD* dihydropyrimidine dehydrogenase polymorphism, *TMB* Tumour mutational burden.

^a^Count (Percentage of column total).

Reported reasons for why recommendations were not followed are summarised in Table 3 and Supplementary Table 12. For 17.8% of recommendations (128/718) participants were either deceased, or not fit/suitable for treatment. For 51.9% of recommendations (373/718), it was not appropriate to change treatment at that time (‘No change to treatment’, ‘Not relevant’, ‘No disease’). It is possible that WGS results could change future management beyond the follow-up of this study for a proportion of these participants.

## Discussion

### Principal findings

The 100,000 Genomes Project was a landmark initiative in the United Kingdom, evaluating the feasibility and benefits of WGS in cancers and rare diseases. This report summarises the experience of WGS for cancer patients who participated in the Project within the West Midlands GMC, the GMC that had recruited the most participants for the Project and covering one of the most diverse populations in the country (Fig. 1). We demonstrated rapid improvement in recruitment and the quality of the samples received during the Project (Fig. 2; Supplementary Fig. 3). Unsurprisingly, the exponential increase in recruitment was accompanied by an increase of other sample issues, including insufficient samples. Of the 4851 sample sets included in the study, 3173 were successfully sequenced, representing 3067 participants of disparate cancer types.

Approximately 60% of participants (1811/3067; 59.0%) were clinically excluded as GTAB discussion would not have changed clinical management (including those who had died, with early-stage cancers or poor fitness). This rate of pre-GTAB exclusion varied significantly between cancer types (Table 1 and Supplementary Table 8), especially in cancers that present with diagnostic challenges or poor fitness at presentation, such as cancer of unknown primary and lung cancer [20, 21]. Conversely, the high rate of exclusion of breast and colorectal cancer participants likely represents a high proportion of curative intent treatment at presentation.

The 100,000 genome project adopted an unselected approach to sequencing, and did not exclude patients based on stage, grade or prognosis. Universal sequencing for cancer patients provides opportunities, including identification of otherwise unrecognised germline variants and contributing to the understanding of the biology of malignancy [6]. However, as demonstrated by our data, a potential weakness of this strategy is that a significant proportion of patients will have sequencing that will not change their cancer management. As a result, the approach presented here may under-estimates the clinical utility of WGS when compared with an approach applied strictly to patients with a permissible performance status and advanced disease.

Six in ten participants discussed at GTAB had recommended actions (59.1%, 548/928); four in ten participants discussed had potential treatments or clinical trials recommended to them (40.4%, 377/933; Table 2), including in cancers with limited therapeutic options such as glioma (50%, 34/68) and hepatobiliary cancers (49%, 50/102). Unfortunately, only a small proportion of potential treatments or clinical trials were followed at the time of last follow-up (5.1%, 25/491). Whilst this is clearly disappointing, it is consistent with reported rates of involvement of cancer patients in clinical trials [22], which is further reduced by socioeconomic deprivation and in ethnic minority groups [23]. An additional challenge for our population was likely presented by the COVID-19 pandemic [24], which may have restricted access to clinical trials and novel therapies. It is possible that with longer follow-up more of our participants may access stratified medicine options based on the WGS data, particularly at times of disease relapse or recurrence.

### Results in the context of what is known

The Project is the largest population-based genomics medicine trial with an overarching aim to introduce WGS within a publicly funded health system. Initial studies using the data from the Project have focused on the genomics and clinical features of specific cancer types [25–28]. Others have explored the potential clinical utility in rare cancer types, including sarcoma and cancers in children [4, 25]. The recently published pan-cancer evaluation of successfully sequenced specimens [6] has used data linkage approach to explore the relationships between tumour genotypes with associated survival. However, it lacked data associated with the clinical decision-making process, nor possible outcomes from patients’ perspectives to support informed shared decision making (Fig. 1). The small but important group of patients with cancers of unknown primaries and haematological cancers were also excluded.

Prendergast and colleagues reported similar rates of WGS being performed from sarcomas (62%) [25]. Prior chemotherapies [25, 29], sample types and disease-specific challenges (e.g., low-grade disease with few cells, or high-grade disease with necrosis) were previously associated with higher failure rates of genetic testing. In addition, treatment-pathway-related factors, such as timing of referral (post-diagnosis) and out-of-hours surgery, have been reported to influence success rates [25].

Similar to a recent report on childhood cancers [4], in which 1 in 5 (22.2%; 8/36) participants undergoing WGS were recommended novel therapeutic opportunities, 3 in 10 (29.8%; 252/846) of our participants whose results were discussed at GTABs were recommended potential treatments or clinical trials (Table 2).

### Strengths and limitations

This study evaluated the clinical processes supporting the implementation of WGS during the Project. It has highlighted the barriers for all cancer types, variations in success rates of WGS and the associated availability of therapeutic opportunities between cancer types. This report complemented the recently published pan-cancer mutational characteristics of the Project [6] with GTAB outcomes of individual participants to demonstrate how WGS has influenced clinical decision making.

Although we identified improvements of quality control during the Project, it is unclear whether any pathway changes were implemented (e.g., removal of formalin from theatre), as previously described [25, 30]. The lack of detailed demographic data, histological subtypes and details of prior treatments precluded further evaluation of risk factors associated with WGS failure and potential strategies to maximise its potential clinical utility.

Our data have not formally evaluated the frequency of diagnosis modifications based on WGS, although and no recorded outcomes from GTAB suggested that diagnosis had been altered. Respectively, 3.0% and 16.7% participants with sarcomas [25] and childhood cancers [4] had their diagnoses refined or changed by their WGS results.

A significant disadvantage of the study was the turnaround time for WGS, caused by the evolution of infrastructure needed to deliver it. However, time to sequencing fell from 16 weeks at the beginning of the Project to 4 weeks at the end, suggesting that building infrastructure and experience facilitates more efficient delivery of WGS. Another weakness of our analysis is that clinical data such as cancer stage was not collated, we are therefore unable to provide a data on the relative utility of WGS in patients with advanced disease.

### Perspectives on WGS

It is important to consider whether WGS is the optimal strategy for universal cancer sequencing. WGS provides comprehensive genomic sequencing, representing a more diagnostically rich approach [31, 32]. In addition, the importance of rich WGS data for cancer research must not be understated [6]. WGS offers the potential to institute standardised pan-cancer sample processing and bioinformatics pipelines, which is attractive for a nationalised healthcare system. A potential disadvantage of WGS is that this data risks overwhelming clinical teams with unexpected results. In the 100,000 Genomes project, this was offset by use of virtual panels, which were then interpreted within the clinical context by GTAB teams. Unlike traditional panel-based sequencing, virtual panels may evolve with scientific understanding [33], allowing for re-analysis of samples, and for evolution of testing to meet service requirements.

Disadvantages of WGS must be acknowledged, with one drawback being time to results and cost-effectiveness compared to alternate technologies [34–36]. Whole-exome or panel sequencing could offer a route to quicker turnaround times, with more rapid turnaround of results potentially allow more patients to access therapies before clinical deterioration. However, as shown here, time to sequencing does fall with establishment of infrastructure for WGS within a healthcare system. A further important consideration is that WGS necessitates either sequencing at either lower depth per gene or committing to prolonged sequencing time [36]. Finally, WGS is poorly validated in FFPE. Successful sequencing is therefore dependent on fresh or fresh-frozen sample, with extraction of relatively large amounts of high-quality DNA [9]. A successful WGS programme therefore requires establishment of robust sampling protocols, with cross-team collaboration [9]. However, the success of the 100,000 Genomes Project demonstrates the feasibility of establishing such a programme at a national level. Clearly the ideal sequencing modality for cancer patients will be influenced by the healthcare system, resources and local expertise.

### Clinical implications

This study provided an estimate of overall rates of successful WGS that led to clinically actionable recommendations. This will improve counselling of patients in a population-based setting with a mixture of different histopathology pathways from both secondary and tertiary hospitals. Notably, recommendations for potential therapeutic options were made in 49.0% (50/102) of participants with hepato-pancreatico-biliary cancers discussed at GTAB and 50% (34/68) of adult participants with glioma discussed at GTAB. This suggests that WGS may provide therapeutic avenues for patients with currently very limited therapeutic options. Targeting these cancer types to establish robust molecular profiling pathways will help achieve potential therapeutic benefits of WGS. In contrast, the management of CUP remains an unmet need. Only one participant with CUP had a potential therapeutic option suggested by GTAB. Of 17 participants recruited, 10 (58.8%) died or were clinically excluded before results interpretation and 5 (29.4%) had no actionable variant. A standardised pathway is needed to expediate diagnosis and management in this challenging group.

It is acknowledged that the genes within Domain 1 are broad, containing genes that are not classically viewed as clinically actionable with current standard of care therapy options. However, these domains have been selected based on expert consensus [10]. Identifying patients with genetic variants that allows matching of patients to stratified medicine trials, providing potential additional treatment lines and providing an opportunity to increase recruitment to these valuable trials.

### Research implications

Previous work highlighted the optimistic view of participants and health professionals on the potential of WGS to improve therapeutic options [37–39]. However, the lack of matched agents with targets remains a hurdle. Careful comparisons of key molecular pathways will generate new hypotheses to evaluate the benefits of targeted therapy in different cancer types. Expanding access to stratified medicine through clinical trials is clearly a priority. For cancer types that have high clinically actionable variant rates and few therapeutic/clinical trial options, tumour agnostic trials are urgently needed to improve outcomes. Whilst large multi-agent, multi-marker studies have been performed, in many cancers the ability to match agents to specific genotypes remains limited. The Cancer Research UK funded DETERMINE study will undertake genotype matched therapy in rare tumours where the therapy is not licensed for that indication to maximise potential benefits of WGS (Clinical Trial registration ID: NCT05722886).

Further research is needed to systematically test methods for improving WGS pathways and implementation of GTAB recommendations. Facilitating use of diagnostic pre-treatment specimens that yield good quality nucleic acids is crucial for implementation of genomics [25, 29]. Moreover, evaluation of patient characteristics (including socioeconomical factors, frailty and ethnicity) will identify good practice and barriers to implementation of genomics in different cancers.

## Conclusions

The Project has established essential National infrastructure and local experience to deliver WGS. Even with an unselected WGS approach, clinical recommendations were made in the majority of participants with interpreted results (522/933; 59.2%; Table 2). Systematic evaluations of genomics medicine pathways in a clinically relevant timeframe will be essential to maximise benefits for patients.

## Supplementary information

Supplementary Material

## Data Availability

The data of the 100,000 Genomes Project can be accessed through a dedicated research environment. Individuals or institutions can join the Genomics England Clinical Interpretation Partnership (GeCIP) to become a member and apply for access to relevant data for research. Further analysis of the data included in this report can be accessed via the corresponding author on a bona fide collaborative basis.

## References

[1] Bailey, Meyerson WU MH, Dursi LJ, Wang L-B, Dong G, Liang W-W, et al. Retrospective evaluation of whole exome and genome mutation calls in 746 cancer samples. Nat Commun. 2020;11:4748. 32958763 10.1038/s41467-020-18151-y PMC7505971

[2] Investigators GPP, Smedley D, Smith KR, Martin A, Thomas EA, McDonagh EM, et al. 100,000 genomes pilot on rare-disease diagnosis in health care - preliminary report. N. Engl J Med. 2021;385:1868–80. 34758253 10.1056/NEJMoa2035790 PMC7613219

[3] Schon KR, Horvath R, Wei W, Calabrese C, Tucci A, Ibanez K, et al. Use of whole genome sequencing to determine genetic basis of suspected mitochondrial disorders: cohort study. BMJ. 2021;375:e066288. 34732400 10.1136/bmj-2021-066288 PMC8565085

[4] Trotman J, Armstrong R, Firth H, Trayers C, Watkins J, Allinson K, et al. The NHS England 100,000 Genomes Project: feasibility and utility of centralised genome sequencing for children with cancer. Br J Cancer. 2022;127:137–44. 35449451 10.1038/s41416-022-01788-5 PMC9276782

[5] Degasperi A, Zou X, Amarante TD, Martinez-Martinez A, Koh GCC, Dias JML, et al. Substitution mutational signatures in whole-genome-sequenced cancers in the UK population. Science. 2022;376:abl9283. 10.1126/science.abl9283 PMC7613262 35949260

[6] Sosinsky A, Ambrose J, Cross W, Turnbull C, Henderson S, Jones L, et al. Insights for precision oncology from the integration of genomic and clinical data of 13,880 tumors from the 100,000 Genomes Cancer Programme. Nat Med. 2024;30:279–89. 38200255 10.1038/s41591-023-02682-0 PMC10803271

[7] Barwell JG, O’Sullivan RBG, Mansbridge LK, Lowry JM, Dorkins HR. Challenges in implementing genomic medicine: the 100,000 Genomes Project. J Transl Genetics Genom. 2018;2:1–10.

[8] Barwell J, Snape K, Wedderburn S. The new genomic medicine service and implications for patients. Clin Med. 2019;19:273–7. 10.7861/clinmedicine.19-4-273 PMC6752257 31308102

[9] Berner AM, Morrissey GJ, Murugaesu N. Clinical analysis of whole genome sequencing in cancer patients. Curr Genet Med Rep. 2019;7:136–43.

[10] Martin AR, Williams E, Foulger RE, Leigh S, Daugherty LC, Niblock O, et al. PanelApp crowdsources expert knowledge to establish consensus diagnostic gene panels. Nat Genet. 2019;51:1560–5. 31676867 10.1038/s41588-019-0528-2

[11] Richards S, Aziz N, Bale S, Bick D, Das S, Gastier-Foster J, et al. Standards and guidelines for the interpretation of sequence variants: a joint consensus recommendation of the American College of Medical Genetics and Genomics and the Association for Molecular Pathology. Genet Med. 2015;17:405–23. 25741868 10.1038/gim.2015.30 PMC4544753

[12] Landrum MJ, Lee JM, Riley GR, Jang W, Rubinstein WS, Church DM, et al. ClinVar: public archive of relationships among sequence variation and human phenotype. Nucleic Acids Res. 2014;42:D980–D5. 24234437 10.1093/nar/gkt1113 PMC3965032

[13] Vandenbroucke JP, von Elm E, Altman DG, Gøtzsche PC, Mulrow CD, Pocock SJ, et al. Strengthening the Reporting of Observational Studies in Epidemiology (STROBE): explanation and elaboration. PLoS Med. 2007;4:e297. 17941715 10.1371/journal.pmed.0040297 PMC2020496

[14] Strickler JH, Hanks BA, Khasraw M. Tumor mutational burden as a predictor of immunotherapy response: is more always better? Clin Cancer Res. 2021;27:1236–41. 33199494 10.1158/1078-0432.CCR-20-3054 PMC9912042

[15] Litchfield K, Reading JL, Puttick C, Thakkar K, Abbosh C, Bentham R, et al. Meta-analysis of tumor- and T cell-intrinsic mechanisms of sensitization to checkpoint inhibition. Cell. 2021;184:596–614.e14. 33508232 10.1016/j.cell.2021.01.002 PMC7933824

[16] Jardim DL, Goodman A, de Melo Gagliato D, Kurzrock R. The challenges of tumor mutational burden as an immunotherapy biomarker. Cancer Cell. 2021;39:154–73. 33125859 10.1016/j.ccell.2020.10.001 PMC7878292

[17] Yarchoan M, Albacker LA, Hopkins AC, Montesion M, Murugesan K, Vithayathil TT, et al. PD-L1 expression and tumor mutational burden are independent biomarkers in most cancers. JCI Insight. 2019;4:e126908. 10.1172/jci.insight.126908 PMC6482991 30895946

[18] Izar B, Tirosh I, Stover EH, Wakiro I, Cuoco MS, Alter I, et al. A single-cell landscape of high-grade serous ovarian cancer. Nat Med. 2020;26:1271–9. 32572264 10.1038/s41591-020-0926-0 PMC7723336

[19] Gallagher PF, Naylor G, Bashir S, Yan X, Burke D, Plummer ER, et al. A CRUK first-in-human phase I trial of LY3143921, a novel CDC7 inhibitor, in patients with advanced solid tumors. Am Soc Clin Oncol. 2022;40:3103.

[20] Adair RA, Scott KJ, Fraser S, Errington-Mais F, Pandha H, Coffey M, et al. Cytotoxic and immune-mediated killing of human colorectal cancer by reovirus-loaded blood and liver mononuclear cells. Int J Cancer. 2013;132:2327–38. 23114986 10.1002/ijc.27918 PMC3891508

[21] Salloum RG, Smith TJ, Jensen GA, Lafata JE. Survival among non-small cell lung cancer patients with poor performance status after first line chemotherapy. Lung Cancer. 2012;77:545–9. 22633939 10.1016/j.lungcan.2012.04.019 PMC3423534

[22] Unger JM, Hershman DL, Till C, Minasian LM, Osarogiagbon RU, Fleury ME, et al. When offered to participate”: a systematic review and meta-analysis of patient agreement to participate in cancer clinical trials. JNCI: J Natl Cancer Inst. 2021;113:244–57. 33022716 10.1093/jnci/djaa155 PMC7936064

[23] Sharrocks K, Spicer J, Camidge D, Papa S. The impact of socioeconomic status on access to cancer clinical trials. Br J cancer. 2014;111:1684–7. 25093493 10.1038/bjc.2014.108 PMC4453719

[24] Nanton V, Bryan RT, Pope AM, Hughes A, Jefferson K, Catto JW, et al. Boosting and broadening recruitment to UK cancer trials: towards a blueprint for action. BMJ Oncol. 2023;2:e000092. 10.1136/bmjonc-2023-000092 PMC11235001 39886495

[25] Prendergast SC, Strobl AC, Cross W, Pillay N, Strauss SJ, Ye H, et al. Sarcoma and the 100,000 Genomes Project: our experience and changes to practice. J Pathol Clin Res. 2020;6:297–307. 32573957 10.1002/cjp2.174 PMC7578291

[26] Yngvadottir B, Andreou A, Bassaganyas L, Larionov A, Cornish AJ, Chubb D, et al. Frequency of pathogenic germline variants in cancer susceptibility genes in 1336 renal cell carcinoma cases. Hum Mol Genet. 2022;31:3001–11. 35441217 10.1093/hmg/ddac089 PMC9433729

[27] Robbe P, Ridout KE, Vavoulis DV, Dreau H, Kinnersley B, Denny N, et al. Whole-genome sequencing of chronic lymphocytic leukemia identifies subgroups with distinct biological and clinical features. Nat Genet. 2022;54:1675–89. 36333502 10.1038/s41588-022-01211-y PMC9649442

[28] Lomas OC, Gooding S, Cabes M, Dreau H, Wilson E, Polzella P, et al. Validation of clinical-grade whole genome sequencing reproduces cytogenetic analysis and identifies mutational landscape in newly-diagnosed multiple myeloma patients: A pilot study from the 100,000 Genomes Project. EJHaem. 2021;2:809–12. 35845211 10.1002/jha2.276 PMC9175844

[29] Chandrasekaran D, Sobocan M, Blyuss O, Miller RE, Evans O, Crusz SM, et al. Implementation of multigene germline and parallel somatic genetic testing in epithelial ovarian cancer: SIGNPOST study. Cancers. 2021;13:4344. 10.3390/cancers13174344 PMC8431198 34503154

[30] Jones L, Craig C. Tissue handling for molecular pathology. RCPath Bull. 2017;180:266–8.

[31] Lionel AC, Costain G, Monfared N, Walker S, Reuter MS, Hosseini SM, et al. Improved diagnostic yield compared with targeted gene sequencing panels suggests a role for whole-genome sequencing as a first-tier genetic test. Genet Med. 2018;20:435–43. 28771251 10.1038/gim.2017.119 PMC5895460

[32] Meienberg J, Bruggmann R, Oexle K, Matyas G. Clinical sequencing: is WGS the better WES? Hum Genet. 2016;135:359–62. 26742503 10.1007/s00439-015-1631-9 PMC4757617

[33] Robertson AJ, Tran K, Patel C, Sullivan C, Stark Z, Waddell N. Evolution of virtual gene panels over time and implications for genomic data re-analysis. Genet Med Open. 2023;1:100820. 10.1016/j.gimo.2023.100820 PMC11613599 39669234

[34] Schwarze K, Buchanan J, Taylor JC, Wordsworth S. Are whole-exome and whole-genome sequencing approaches cost-effective? A systematic review of the literature. Genet Med. 2018;20:1122–30. 29446766 10.1038/gim.2017.247

[35] Laskin J, Jones S, Aparicio S, Chia S, Ch’ng C, Deyell R, et al. Lessons learned from the application of whole-genome analysis to the treatment of patients with advanced cancers. Mol Case Stud. 2015;1:a000570. 10.1101/mcs.a000570 PMC4850882 27148575

[36] Koboldt DC. Best practices for variant calling in clinical sequencing. Genome Med. 2020;12:91. 33106175 10.1186/s13073-020-00791-w PMC7586657

[37] Ballard LM, Horton RH, Dheensa S, Fenwick A, Lucassen AM. Exploring broad consent in the context of the 100,000 Genomes Project: a mixed methods study. Eur J Hum Genet. 2020;28:732–41. 31919452 10.1038/s41431-019-0570-7 PMC7253456

[38] Sanderson SC, Hill M, Patch C, Searle B, Lewis C, Chitty LS. Delivering genome sequencing in clinical practice: an interview study with healthcare professionals involved in the 100 000 Genomes Project. BMJ Open. 2019;9:e029699. 31685495 10.1136/bmjopen-2019-029699 PMC6858183

[39] Sanderson SC, Lewis C, Hill M, Peter M, McEntagart M, Gale D, et al. Decision-making, attitudes, and understanding among patients and relatives invited to undergo genome sequencing in the 100,000 Genomes Project: A multisite survey study. Genet Med. 2022;24:61–74. 34906473 10.1016/j.gim.2021.08.010

