# Supplementary material for: The potential clinical utility of Whole Genome Sequencing for patients with cancer: evaluation of a regional implementation of the 100,000 Genomes Project
Source: Br J Cancer. 2024 Oct 30;131(11):1805–13. doi: 10.1038/s41416-024-02890-6 (PMC11589591; doi:10.1038/s41416-024-02890-6)
Supplement: Supplementary file 1 — Supplementary Material [file 41416_2024_2890_MOESM1_ESM.docx]

**Supplementary Materials**

**1.1 Supplementary Methods**

**1.1.1 Summarized Protocols for the 100K Genome Project**

**Inclusion and Exclusion Data** *(Summarized and reproduced from ‘Approved List of Cancers and their Eligibility Criteria’)^1^*

***Overview***

- Potential participants not wanting to consent for the study or participate in all aspect of the Project should be excluded
- A patient who has had whole genome sequencing as part of another project should not be recruited to the 100,00 genomes Project (unless otherwise agreed)
- Patients may be recruited in parallel to a clinical trial, provided the clinical trial sample will not be compromised and the sample will not undergo Whole Genome Sequencing (unless otherwise agreed)
- Participants should only be recruited if the Essential sample Data and Core Data will be obtainable
- All participants must be residents of England, Scotland, Northern Ireland or Wales and be under the care of and be followed up by the NHS in England. Those in England and Wales must have an NHS number, and those resident in Scotland must have their country equivalent
- Samples may be from primary lesion or metastasis
- Multiple samples may be accepted from a single patient: synchronous tumours, metastatic and primary sites, samples from different locations within a tumour, samples taken at different time points

***Inclusion:***

- Participants must have a WHO/IARC classification cancer diagnosis (invasive malignancy)
- The specified dataset must be collectable within agreed time scales
- Informed consent
- Previously treated patients are eligible who
  - Present with recurrence of a previously treated tumour (may be local or metastatic recurrence)
  - Have undergone cancer therapy (chemotherapy, hormone therapy, radiotherapy) but progress on treatment
  - Have received neoadjuvant chemotherapy
  - Have undergone cancer therapy (chemotherapy, hormone therapy, radiotherapy) for a previous tumour

***Exclusion***

Ineligible cancer types:

- Cervical, vaginal and vulval carcinoma (aside from melanoma)
- Endocrine malignancies (aside from thyroid cancer)
- Squamous and basal skin cancers
- Malignancy of the placenta or heart
- Malignancy of the male genital tract (aside from prostate, testis, melanoma)
- Benign tumors
- Carcinoma in situ (except bladder)
- Borderline ovarian tumours

***Expanded Haematological Malignancy Eligibility (****Summarised from ‘Updated Haematological Malignancy Eligibility’)^2^*

Eligibility criteria were broadened in 2017 to expand eligibility to more patients with haematological malignancies

***Inclusion:***

- All patients with haematological malignancy with > 40% malignant nuclei on peripheral blood or bone marrow aspirate for whom imminent treatment is planned (including multiple myeloma with > 40% CD138 positive cells following enrichment)
- All patients with acute leukemia (as defined by WHO classification of haematopoietic and lymphoid tumours, 2016) i.e. ≥ 20% blasts or with leukemia defining genetic aberration
- Myelodysplastic syndrome with ≥ 5% blasts
- Patients with Chronic Myeloid Leukemia (CML) who meet one of the following:
  - After 3 months of treatment with a tyrosine kinase inhibitor have BCR-ABL transcript levels <1% (extreme good responder) or > 10% (extreme bad responder) by RQ-PCR
  - Present in accelerated or blast phase (> 10% blasts in bone marrow or peripheral blood)
  - Cytogenetic abnormality in addition to t(9;22) at diagnosis (excluding those where sole cytogenetic abnormlity is a variant transcript)
  - Progress from chronic phase to accelerated or blast phase
- Unclassified or unknown disease (for example: myelodysplastic/myeloproliferative neoplasm overlap syndromes, triple negative myeloproliferative neoplasm (no variant in JAK2 exon 12 or codon 617, CALR exon 9, MPL exon 10), or uncertain diagnosis where clinical presentation does not fit with pathological diagnosis

***Exclusion:***

- Chronic lymphocytic lymphoma or other lymohoproliferative disorder with no planned treatment
- Myelodysplastic syndrome with <5% blasts
- Stable chronic phase chromic myeloid leukemia or other myeloproliferative neoplasms

**1.1.2 Summarized Protocols for the 100K Genome Project**

*(Summarised and reproduced from Sample Handling Guidance^3^)*

***Quality Assurance***

Designated Laboratories were required to have active Clinical Pathology Accredition (UK) limited accreditation and either be accredited or working within the UKAS phasing plan for accredition to ISO 15189. All designated DNA extraction laboratories were required to participate in the UK National External Quality Assurance Scheme (UK NEQAS for molecular genetics / GenQA)

***Sample handling***

Samples were collected and handled according to the Project’s standard operating procedures (Version 4).^3^ Germline and tumour samples were essential for analysis, an additional plasma sample for cfDNA was optional.

Blood was collected for germline samples. Stored DNA, cultured fibroblasts or saliva samples were accepted for germline sample under exceptional circumstances. Fresh-frozen tumour samples could be surgical or biopsy specimens, providing at least 40% of nucleated cells being tumour cells. Under exceptional circumstance, formalin-fixed Paraffin-Embedded (FFPE) samples were accepted.

Any DNA extraction protocol suitable for fresh frozen tissue was permitted within the national 100k genome project. DNA was extracted by using QIASymphony SP in the WMRGL. DNA concentration was measured locally, and re-measured by the UK Biobank and Illumina. 10 µg DNA was required for germline samples. For tumour samples: 2 µg tumour DNA was preferred for PCR-free WGS (1.3 µg was accepted for a single library preparation PCR-free sequencing). PCR amplified sequencing was performed where DNA was insufficient for PCR-free library preparation (minimum input for PCR-amplified sequencing: 500 ng DNA). Samples with insufficient DNA were rejected.

***Sequencing***

Sequencing was performed according to the Project’s standard operating procedures. Library preparation was performed by Illumina (Cambridge, United Kingdom) using TruSeq DNA Nano, TruSeq DNA PCR-Free, or FFPE library preparation kits. Sequencing was performed using HiSeq X resulting in 150 bp paired-end reads. Germline samples were sequenced to ≥ 85 Gb (sequencing quality ≥ 30). Tumour samples were sequenced to ≥ 212.5 Gb, with at least 95% of genome covered at ≥ 15x with well mapped reads (mapping quality > 10 after duplicates discarded)

***Genomic analysis***

Analysis was performed according to the Project’s standard operating procedures. Germline samples were assessed for cross-contamination using VerifyBamID (pass if < 3%); Tumour samples were assessed for cross-contamination using ConPair (pass if < 1%, fail if > 5%). Illumina’s North Star Pipeline (version 2.6.63.23) was used for primary analysis, aligning against GRCH38-Decoy+EBV with ISAAC (03.16.02.19). Small variant calling and tumour-normal subtraction used Strelka (Version 2.4.7). Structural variants (SVs) and long indel (>50 bp) were called using Manta (version 0.28.0). Copy Number Variants (CNVs) were called using Canvas (version 1.3.1).

SNVs were annotated using Cellbase [ENSEMBL (version 90/GRCh38), COSMIC (version v86/GRCh38) and ClinVar (October 2018 release)], reporting only variants which altered canonical transcripts (transcript ablation, splice acceptor variant, splice donor variant, stop gain, frameshift variant, stop loss, start loss, transcript amplification, in-frame insertion, in-frame deletion, in-frame variant, missense variant, splice region variant).

***Stratification of Somatic Variants***

Reported somatic variants were stratified into three domains, according to virtual panels (<https://panelapp.genomicsengland.co.uk/>). Variants in potentially actionable genes were triaged into Domain 1. Potentially actionable genes were defined as genes where small variant have been associated with prognosis, response to therapy or clinical trial entry (actively recruiting or closed UK clinical trials), as determined by GenomOncology. According to this definition, 143 genes were listed as actionable in solid tumours (Supplementary table 1), and 118 genes were defined as actionable in haematological malignancies (Supplementary table 2). Where data was available, actionability at the level of variants was annotated (variant-level actionability). Domain 2 encompassed somatic variants in a panel of 535 cancer-related genes. Cancer-related genes were defined as genes in which variants have been causally implicated in cancer defined by Cancer Gene Census (Supplementary table 3). Domain 3 encompassed variants not included in Domain 1 or 2. Variants were reviewed locally for clinical significance.

***Stratification of Germline Variants***

Germline variants were stratified. Tier 1 variants were determined according to a tumour-type specific virtual genes panels (<https://panelapp.genomicsengland.co.uk/>; Supplementary Table 4). Pathogenic or likely pathogenic alterations were truncating mutations in genes where loss of function is pathogenic, or where variants were listed in ClinVar as pathogenic or likely pathogenic. Variants defined as benign or likely benign in ClinVar were excluded. Domain 3 germline variants were determined according to wider virtual gene panels (<https://panelapp.genomicsengland.co.uk/>; Supplementary Table 5 and 6). Germline samples were also screened for pertinent alterations in DPYD which confer DPYD deficiency and are therefore of pharmacogenomic significance (Supplementary Table 7).

***Assessment of Microsatellite Instability (MSI) and Tumour Mutational Burden (TMB)***

Microsatellite Instability (MSI) was calculated using information entropy metrics, the software assesses all microsatellite sites for evidence of instability relative to a set of baseline normal samples. The proportion of unstable MSI sites to total assessed MSI sites is reported as a sample-level microsatellite score, with a score > 20% being reported as microsatellite instable. Tumour mutational burden was calculated by taking all coding non-synonymous mutations as assessed by annotation by Illumina Isis/Nirvana software then division by the coding region size.

***Further information***

Further information could be obtained from the dedicated webpage of the Project (<https://www.genomicsengland.co.uk/initiatives/100000-genomes-project/documentation>).

***References***

1. Approved List of Cancers and Their Eligibility Criteria (Updated April 2018). Accessed from: <https://www.genomicsengland.co.uk/initiatives/100000-genomes-project/documentation>. Last accessed June 2024.
2. Updated Haematological Malignancy Eligibility (data model v3.2.0) PAR-GUI-050, v0.1. 2018. Accessed from: <https://www.genomicsengland.co.uk/initiatives/100000-genomes-project/documentation>. Last accessed 2 August 2023
3. Genomics England Research Consortium. Sample Handling Guidance (Version 4; document key PAR-GUI-023). 2018. <https://files.genomicsengland.co.uk/forms/Sample-Handling-Guidance-v4.0.pdf>. Last accessed 2 August 2023.
4. Genomics England. Cancer Analysis Technical Information Document (Version 2.0; document key 616). 2020. <https://files.genomicsengland.co.uk/forms/Cancer-Analysis-Technical-Information-Document-v1-11-main.pdf>. Last accessed 3 October 2023
5. Genomics England Research Consortium. Approved List of Cancers and Their Eligibility Criteria <https://www.genomicsengland.co.uk/initiatives/100000-genomes-project/documentation>. Last accessed 3 October 2023

**1.2 Supplementary Tables**

**Supplementary Table 1 (Online only):** Genes considered Actionable in solid tumours 100,000 Genome Project Adapted from Actionable genes in solid tumour small variants v1.11 (Genomics England. <https://www.genomicsengland.co.uk/initiatives/100000-genomes-project/documentation>, last accessed 03.10.2023)

| Gene Name | Gene ID | Gene Name | Gene ID | Gene Name | Gene ID |
| --- | --- | --- | --- | --- | --- |
| AKT1 | ENSG00000142208 | FGF17 | ENSG00000158815 | NBN | ENSG00000104320 |
| AKT2 | ENSG00000105221 | FGF18 | ENSG00000156427 | NF1 | ENSG00000196712 |
| AKT3 | ENSG00000117020 | FGF19 | ENSG00000162344 | NPM1 | ENSG00000181163 |
| ALK | ENSG00000171094 | FGF2 | ENSG00000138685 | NRAS | ENSG00000213281 |
| APC | ENSG00000134982 | FGF20 | ENSG00000078579 | NTRK1 | ENSG00000198400 |
| ARAF | ENSG00000078061 | FGF21 | ENSG00000105550 | NTRK2 | ENSG00000148053 |
| ATM | ENSG00000149311 | FGF22 | ENSG00000070388 | NTRK3 | ENSG00000140538 |
| ATR | ENSG00000175054 | FGF23 | ENSG00000118972 | PALB2 | ENSG00000083093 |
| ATRX | ENSG00000085224 | FGF3 | ENSG00000186895 | PARP1 | ENSG00000143799 |
| BAP1 | ENSG00000163930 | FGF4 | ENSG00000075388 | PARP2 | ENSG00000129484 |
| BARD1 | ENSG00000138376 | FGF5 | ENSG00000138675 | PDGFRA | ENSG00000134853 |
| BRAF | ENSG00000157764 | FGF6 | ENSG00000111241 | PIK3CA | ENSG00000121879 |
| BRCA1 | ENSG00000012048 | FGF7 | ENSG00000140285 | PIK3CB | ENSG00000051382 |
| BRCA2 | ENSG00000139618 | FGF8 | ENSG00000107831 | PIK3CG | ENSG00000105851 |
| BRIP1 | ENSG00000136492 | FGF9 | ENSG00000102678 | PIK3R1 | ENSG00000145675 |
| CDK12 | ENSG00000167258 | FGFR1 | ENSG00000077782 | PIK3R2 | ENSG00000105647 |
| CDKN2A | ENSG00000147889 | FGFR2 | ENSG00000066468 | PMS1 | ENSG00000064933 |
| CEBPA | ENSG00000245848 | FGFR3 | ENSG00000068078 | PMS2 | ENSG00000122512 |
| CHEK1 | ENSG00000149554 | FGFR4 | ENSG00000160867 | PPP2R1A | ENSG00000105568 |
| CHEK2 | ENSG00000183765 | FLT3 | ENSG00000122025 | PPP2R2A | ENSG00000221914 |
| CRKL | ENSG00000099942 | FRS2 | ENSG00000166225 | PTEN | ENSG00000171862 |
| CTNNA1 | ENSG00000044115 | GRB2 | ENSG00000177885 | PTPN11 | ENSG00000179295 |
| CTNNA2 | ENSG00000066032 | HDAC1 | ENSG00000116478 | RAD50 | ENSG00000113522 |
| CTNNA3 | ENSG00000183230 | HDAC2 | ENSG00000196591 | RAD51 | ENSG00000051180 |
| CTNNB1 | ENSG00000168036 | HRAS | ENSG00000174775 | RAD51B | ENSG00000182185 |
| EGFR | ENSG00000146648 | IDH1 | ENSG00000138413 | RAD51C | ENSG00000108384 |
| EMSY | ENSG00000158636 | IRS2 | ENSG00000185950 | RAD51D | ENSG00000185379 |
| ERBB2 | ENSG00000141736 | KIT | ENSG00000157404 | RAD54L | ENSG00000085999 |
| ERBB3 | ENSG00000065361 | KLB | ENSG00000134962 | RAF1 | ENSG00000132155 |
| ESR1 | ENSG00000091831 | KRAS | ENSG00000133703 | RB1 | ENSG00000139687 |
| EZH2 | ENSG00000106462 | MAP2K1 | ENSG00000169032 | RET | ENSG00000165731 |
| FANCA | ENSG00000187741 | MAP2K2 | ENSG00000126934 | RICTOR | ENSG00000164327 |
| FANCB | ENSG00000181544 | MAP2K4 | ENSG00000065559 | RNF43 | ENSG00000108375 |
| FANCC | ENSG00000158169 | MAP3K1 | ENSG00000095015 | ROS1 | ENSG00000047936 |
| FANCD2 | ENSG00000144554 | MAPK1 | ENSG00000100030 | RPTOR | ENSG00000141564 |
| FANCE | ENSG00000112039 | MCPH1 | ENSG00000147316 | SLX4 | ENSG00000188827 |
| FANCF | ENSG00000183161 | MDM2 | ENSG00000135679 | SMARCA4 | ENSG00000127616 |
| FANCG | ENSG00000221829 | MDM4 | ENSG00000198625 | SMARCB1 | ENSG00000099956 |
| FANCI | ENSG00000140525 | MET | ENSG00000105976 | SMO | ENSG00000128602 |
| FANCL | ENSG00000115392 | MLH1 | ENSG00000076242 | SRC | ENSG00000197122 |
| FANCM | ENSG00000187790 | MLH3 | ENSG00000119684 | STAG2 | ENSG00000101972 |
| FGF1 | ENSG00000113578 | MRE11 | ENSG00000020922 | STK11 | ENSG00000118046 |
| FGF10 | ENSG00000070193 | MSH2 | ENSG00000095002 | TP53 | ENSG00000141510 |
| FGF11 | ENSG00000161958 | MSH3 | ENSG00000113318 | TSC1 | ENSG00000165699 |
| FGF12 | ENSG00000114279 | MSH6 | ENSG00000116062 | TSC2 | ENSG00000103197 |
| FGF13 | ENSG00000129682 | MTOR | ENSG00000198793 | WNT5A | ENSG00000114251 |
| FGF14 | ENSG00000102466 | MUTYH | ENSG00000132781 | XRCC1 | ENSG00000073050 |
| FGF16 | ENSG00000196468 | MYC | ENSG00000136997 |  |  |

**Supplementary Table 2 (Online only):** Genes considered Actionable in haematological malignancies 100,000 Genome Project (Taken from Actionable genes in haemonc v1.11). Adapted from Actionable genes in haemonc v1.11 (Genomics England. <https://www.genomicsengland.co.uk/initiatives/100000-genomes-project/documentation>, last accessed 03.10.2023)

| Gene Name | Gene ID | Gene Name | Gene ID | Gene Name | Gene ID |
| --- | --- | --- | --- | --- | --- |
| ABL1 | ENSG00000097007 | FGF17 | ENSG00000158815 | MUTYH | ENSG00000132781 |
| ALK | ENSG00000171094 | FGF18 | ENSG00000156427 | MYC | ENSG00000136997 |
| ASXL1 | ENSG00000171456 | FGF19 | ENSG00000162344 | MYD88 | ENSG00000172936 |
| ATM | ENSG00000149311 | FGF2 | ENSG00000138685 | NBN | ENSG00000104320 |
| ATR | ENSG00000175054 | FGF20 | ENSG00000078579 | NF1 | ENSG00000196712 |
| ATRX | ENSG00000085224 | FGF21 | ENSG00000105550 | NOTCH1 | ENSG00000148400 |
| BARD1 | ENSG00000138376 | FGF22 | ENSG00000070388 | NPM1 | ENSG00000181163 |
| BCOR | ENSG00000183337 | FGF23 | ENSG00000118972 | NRAS | ENSG00000213281 |
| BIRC3 | ENSG00000023445 | FGF3 | ENSG00000186895 | NTRK1 | ENSG00000198400 |
| BRAF | ENSG00000157764 | FGF4 | ENSG00000075388 | NTRK2 | ENSG00000148053 |
| BRCA1 | ENSG00000012048 | FGF5 | ENSG00000138675 | NTRK3 | ENSG00000140538 |
| BRCA2 | ENSG00000139618 | FGF6 | ENSG00000111241 | PALB2 | ENSG00000083093 |
| BRIP1 | ENSG00000136492 | FGF7 | ENSG00000140285 | PARP1 | ENSG00000143799 |
| CBL | ENSG00000110395 | FGF8 | ENSG00000107831 | PARP2 | ENSG00000129484 |
| CCND1 | ENSG00000110092 | FGF9 | ENSG00000102678 | PMS1 | ENSG00000064933 |
| CDK12 | ENSG00000167258 | FGFR1 | ENSG00000077782 | PMS2 | ENSG00000122512 |
| CEBPA | ENSG00000245848 | FGFR2 | ENSG00000066468 | PPP2R1A | ENSG00000105568 |
| CHEK1 | ENSG00000149554 | FGFR3 | ENSG00000068078 | PPP2R2A | ENSG00000221914 |
| CHEK2 | ENSG00000183765 | FGFR4 | ENSG00000160867 | PRPF8 | ENSG00000174231 |
| DNMT3A | ENSG00000119772 | FLT3 | ENSG00000122025 | PTEN | ENSG00000171862 |
| EMSY | ENSG00000158636 | GATA1 | ENSG00000102145 | PTPN11 | ENSG00000179295 |
| ETV6 | ENSG00000139083 | HDAC1 | ENSG00000116478 | RAD50 | ENSG00000113522 |
| EZH2 | ENSG00000106462 | HDAC2 | ENSG00000196591 | RAD51 | ENSG00000051180 |
| FANCA | ENSG00000187741 | HRAS | ENSG00000174775 | RAD51B | ENSG00000182185 |
| FANCB | ENSG00000181544 | IDH1 | ENSG00000138413 | RAD51C | ENSG00000108384 |
| FANCC | ENSG00000158169 | IDH2 | ENSG00000182054 | RAD51D | ENSG00000185379 |
| FANCD2 | ENSG00000144554 | IKZF1 | ENSG00000185811 | RAD54L | ENSG00000085999 |
| FANCE | ENSG00000112039 | JAK2 | ENSG00000096968 | RUNX1 | ENSG00000159216 |
| FANCF | ENSG00000183161 | KIT | ENSG00000157404 | SETBP1 | ENSG00000152217 |
| FANCG | ENSG00000221829 | KRAS | ENSG00000133703 | SF3B1 | ENSG00000115524 |
| FANCI | ENSG00000140525 | MCPH1 | ENSG00000147316 | SLX4 | ENSG00000188827 |
| FANCL | ENSG00000115392 | MDM2 | ENSG00000135679 | SRSF2 | ENSG00000161547 |
| FANCM | ENSG00000187790 | MDM4 | ENSG00000198625 | STAG2 | ENSG00000101972 |
| FGF1 | ENSG00000113578 | MLH1 | ENSG00000076242 | TET2 | ENSG00000168769 |
| FGF10 | ENSG00000070193 | MLH3 | ENSG00000119684 | TP53 | ENSG00000141510 |
| FGF11 | ENSG00000161958 | MPL | ENSG00000117400 | U2AF1 | ENSG00000160201 |
| FGF12 | ENSG00000114279 | MRE11 | ENSG00000020922 | XRCC1 | ENSG00000073050 |
| FGF13 | ENSG00000129682 | MSH2 | ENSG00000095002 | ZRSR2 | ENSG00000169249 |
| FGF14 | ENSG00000102466 | MSH3 | ENSG00000113318 |  |  |
| FGF16 | ENSG00000196468 | MSH6 | ENSG00000116062 |  |  |

**Supplementary Table 3 (online only):** Cancer-related genes that have been causally implicated in cancer as defined by Cancer Gene Census. Somatic variants in these genes were triaged into Domain 2. Adapted from Cancer Census Genes v.10 (Genomics England. <https://www.genomicsengland.co.uk/initiatives/100000-genomes-project/documentation>, last accessed 03.10.2023)

| Cancer-Related Genes | | | | | | | | |
| --- | --- | --- | --- | --- | --- | --- | --- | --- |
| ABI1 | CAMTA1 | DDX10 | FOXP1 | KDM6A | MSN | PDGFRB | RPL10 | TCF7L2 |
| ABL1 | CANT1 | DDX3X | FSTL3 | KDR | MTCP1 | PER1 | RPL22 | TCL1A |
| ABL2 | CARD11 | DDX5 | FUBP1 | KDSR | MTOR | PHF6 | RPL5 | TERT |
| ACKR3 | CARS | DDX6 | FUS | KEAP1 | MUC1 | PHOX2B | RPN1 | TET1 |
| ACSL3 | KNL1 | DEK | GAS7 | KIF5B | MYB | PICALM | RSPO2 | TET2 |
| ACVR1 | CASP8 | DICER1 | GATA1 | KIT | MYC | PIK3CA | RSPO3 | TFE3 |
| ACVR2A | CBFA2T3 | DNAJB1 | GATA2 | KLF4 | MYCL | PIK3CB | RUNX1 | TFEB |
| AFF1 | CBFB | DNM2 | GATA3 | KLF6 | MYCN | PIK3R1 | RUNX1T1 | TFG |
| AFF3 | CBL | DNMT3A | GNA11 | KLK2 | MYD88 | PIM1 | SALL4 | TGFBR2 |
| AFF4 | CBLB | DROSHA | GNAQ | KMT2A | MYH11 | PLAG1 | SDC4 | TLX1 |
| AKT1 | CBLC | EBF1 | GNAS | KMT2C | MYH9 | PLCG1 | SDHA | TLX3 |
| AKT2 | CCDC6 | EGFR | GOLGA5 | KMT2D | MYO5A | PML | SET | TMPRSS2 |
| ALK | CCNB1IP1 | EIF3E | GOPC | KRAS | MYOD1 | POLD1 | SETBP1 | TNFAIP3 |
| AMER1 | CCND1 | EIF4A2 | GPHN | KTN1 | NAB2 | POLE | SETD2 | TNFRSF14 |
| APC | CCND2 | ELF4 | GRIN2A | LASP1 | NCOA1 | POLQ | SF3B1 | TNFRSF17 |
| AR | CCND3 | ELK4 | H3F3A | LATS1 | NCOA2 | POT1 | SFPQ | TOP1 |
| ARHGAP26 | CCNE1 | ELL | H3F3B | LATS2 | NCOA4 | POU2AF1 | SFRP4 | TP53 |
| ARHGEF12 | CD274 | EML4 | HERPUD1 | LCK | NCOR1 | POU5F1 | SH2B3 | TP63 |
| ARID1A | CD74 | EP300 | HEY1 | LEF1 | NCOR2 | PPARG | SH3GL1 | TPM3 |
| ARID1B | CD79A | EPAS1 | HIF1A | LIFR | NDRG1 | PPFIBP1 | SIX1 | TPM4 |
| ARID2 | CD79B | EPS15 | HIP1 | LMNA | NF1 | PPM1D | SLC34A2 | TPR |
| ARNT | CDC73 | ERBB2 | HIST1H3B | LMO1 | NF2 | PPP2R1A | SLC45A3 | TRAF7 |
| ASPSCR1 | CDH1 | ERBB3 | HIST1H4I | LMO2 | NFATC2 | PPP6C | SMAD2 | TRIM24 |
| ASXL1 | CDH11 | ERBB4 | HLA-A | LPP | NFE2L2 | PRCC | SMAD3 | TRIM27 |
| ATF1 | CDK12 | ERC1 | HLF | LRIG3 | NFIB | PRDM1 | SMAD4 | TRIM33 |
| ATIC | CDK6 | ERG | HMGA1 | LRP1B | NFKB2 | PRDM16 | SMARCA4 | TRIP11 |
| ATM | CDKN1B | ESR1 | HMGA2 | LYL1 | NFKBIE | PREX2 | SMARCB1 | TRRAP |
| ATP1A1 | CDKN2A | ETNK1 | HNF1A | LZTR1 | NIN | PRKACA | SMARCD1 | TSC1 |
| ATP2B3 | CDKN2C | ETV1 | HNRNPA2B1 | MAF | NKX2-1 | PRKAR1A | SMO | TSC2 |
| ATR | CDX2 | ETV4 | HOOK3 | MAFB | NONO | PRRX1 | SND1 | TSHR |
| ATRX | CEBPA | ETV5 | HOXA11 | MALT1 | NOTCH1 | PSIP1 | SOCS1 | U2AF1 |
| AXIN1 | CHCHD7 | ETV6 | HOXA13 | MAML2 | NOTCH2 | PTCH1 | SOX2 | UBR5 |
| AXIN2 | CHD4 | EWSR1 | HOXA9 | MAP2K1 | NPM1 | PTEN | SPEN | USP6 |
| B2M | CIC | EZH2 | HOXC11 | MAP2K2 | NR4A3 | PTK6 | SPOP | USP8 |
| BAP1 | CIITA | EZR | HOXC13 | MAP2K4 | NRAS | PTPN11 | SRC | VHL |
| BARD1 | CLIP1 | FAM46C | HOXD11 | MAP3K1 | NRG1 | PTPN13 | SRSF2 | NSD2 |
| BAX | CLTC | FAS | HOXD13 | MAP3K13 | NSD1 | PTPRB | SRSF3 | NSD3 |
| BCL10 | CLTCL1 | FAT1 | HRAS | MAPK1 | NT5C2 | PTPRC | SS18 | WIF1 |
| BCL11A | CNBP | FAT4 | HSP90AA1 | MAX | NTRK1 | PTPRK | SS18L1 | WT1 |
| BCL11B | CNOT3 | FBXO11 | HSP90AB1 | MDM2 | NTRK3 | PTPRT | SSX1 | WWTR1 |
| BCL2 | CNTRL | FBXW7 | IDH1 | MDM4 | NUMA1 | QKI | SSX2 | XPO1 |
| BCL3 | COL1A1 | FCGR2B | IDH2 | MECOM | NUP214 | RABEP1 | SSX4 | YWHAE |
| BCL6 | COL2A1 | FCRL4 | IKBKB | MED12 | NUP98 | RAC1 | STAG2 | ZBTB16 |
| BCL7A | CREB1 | FES | IKZF1 | MEN1 | NUTM1 | RAD21 | STAT3 | ZFHX3 |
| BCL9 | CREB3L1 | FEV | IL2 | MET | NUTM2A | RAD51B | STAT5B | ZMYM2 |
| BCL9L | CREB3L2 | FGFR1 | IL21R | MITF | NUTM2B | RAF1 | STAT6 | PATZ1 |
| BCOR | CREBBP | FGFR1OP | IL6ST | MKL1 | OLIG2 | RANBP2 | STIL | ZNF331 |
| BCORL1 | CRLF2 | FGFR2 | IL7R | MLF1 | P2RY8 | RAP1GDS1 | STK11 | ZNF384 |
| BCR | CRTC1 | FGFR3 | IRF4 | MLH1 | PAFAH1B2 | RARA | STRN | ZNF521 |
| BIRC3 | CRTC3 | FGFR4 | IRS4 | MLLT1 | PAX3 | RB1 | SUFU | ZRSR2 |
| BRAF | CSF3R | FHIT | ITK | MLLT10 | PAX5 | RBM10 | SUZ12 | IGH |
| BRCA1 | CTCF | FIP1L1 | JAK1 | MLLT11 | PAX7 | RBM15 | SYK | IGK |
| BRCA2 | CTNNB1 | FLI1 | JAK2 | MLLT3 | PAX8 | REL | TAF15 | IGL |
| BRD3 | CUX1 | FLT3 | JAK3 | AFDN | PBRM1 | RET | TAL1 | TRA |
| BRD4 | CXCR4 | FLT4 | JUN | MLLT6 | PBX1 | RHOA | TAL2 | TRB |
| BTG1 | CYLD | FOXA1 | KAT6A | MN1 | PCM1 | RHOH | TBL1XR1 | TRD |
| BTK | DAXX | FOXL2 | KAT6B | MPL | PDCD1LG2 | RMI2 | TBX3 |  |
| WDCP | DCTN1 | FOXO1 | KCNJ5 | MSH2 | PDE4DIP | RNF213 | TCEA1 |  |
| CACNA1D | DDIT3 | FOXO3 | KDM5A | MSH6 | PDGFB | RNF43 | TCF12 |  |
| CALR | DDR2 | FOXO4 | KDM5C | MSI2 | PDGFRA | ROS1 | TCF3 |  |

**Supplementary Table 4 (online only):** Cancer site-specific susceptibility genes for which pathogenic or likely pathogenic variants were triaged as Tier 1 germline findings (Adapted from: <http://panelapp.genomicsengland.co.uk>)

| **Bladder** | **Brain** | **Breast** | **Colorectal** | **Endometrial** | **H&N** | **Melanoma** | **Neuroendocrine** | **Ovarian** | **Prostate** | **Renal** | **Sarcoma** | **Thyroid** | **Upper GI** |
| --- | --- | --- | --- | --- | --- | --- | --- | --- | --- | --- | --- | --- | --- |
| *MLH1* | *APC* | *BRCA1* | *APC* | *FH* | *BRIP1* | *BAP1* | *CDKN1B* | *BRCA1* | *BRCA2* | *FH* | *MTAP* | *CDKN1B* | *MLH1* |
| *MSH2* | *ATM* | *BRCA2* | *BMPR1A* | *MLH1* | *FANCA* | *CDK4* | *FH* | *BRCA2* | *MLH1* | *FLCN* | *PDGFRA* | *PRKAR1A* | *MSH2* |
| *MSH6* | *MLH1* | *PALB2* | *MLH1* | *MSH2* | *FANCB* | *CDKN2A* | *MAX* | *BRIP1* | *MSH2* | *MET* | *RECQL4* | *PTEN* | *MSH6* |
| *PMS2* | *MSH2* | *TP53* | *MSH2* | *MSH6* | *FANCC* |  | *MEN1* | *MLH1* | *MSH6* | *MLH1* | *SQSTM1* | *RET* | *PMS2* |
|  | *MSH6* | *ATRIP* | *MSH6* | *PMS2* | *FANCD2* |  | *RET* | *MSH2* | *PMS2* | *MSH2* | *T* | *WRN* |  |
|  | *PMS2* | *CDH1* | *MUTYH* | *PTEN* | *FANCE* |  | *SDHA* | *MSH6* |  | *MSH6* | *TP53* |  |  |
|  | *TP53* | *PTEN* | *NTHL1* |  | *FANCF* |  | *SDHAF2* | *RAD51C* |  | *PMS2* | *BUB1B* |  |  |
|  |  |  | *PMS2* |  | *FANCG* |  | *SDHB* | *RAD51D* |  | *PTEN* | *CDKN1C* |  |  |
|  |  |  | *POLD1* |  | *FANCI* |  | *SDHC* | *PMS2* |  | *SDHB* | *EXT1* |  |  |
|  |  |  | *POLE* |  | *FANCL* |  | *SDHD* |  |  | *VHL* | *EXT2* |  |  |
|  |  |  | *PTEN* |  | *SLX4* |  | *TMEM127* |  |  |  | *FH* |  |  |
|  |  |  | *SMAD4* |  | *FANCM* |  | *VHL* |  |  |  | *HRAS* |  |  |
|  |  |  | *STK11* |  |  |  |  |  |  |  | *KIT* |  |  |
|  |  |  |  |  |  |  |  |  |  |  | *MLH1* |  |  |
|  |  |  |  |  |  |  |  |  |  |  | *MSH2* |  |  |
|  |  |  |  |  |  |  |  |  |  |  | *MSH6* |  |  |
|  |  |  |  |  |  |  |  |  |  |  | *NBN* |  |  |
|  |  |  |  |  |  |  |  |  |  |  | *NF1* |  |  |
|  |  |  |  |  |  |  |  |  |  |  | *PMS2* |  |  |
|  |  |  |  |  |  |  |  |  |  |  | *RB1* |  |  |
|  |  |  |  |  |  |  |  |  |  |  | *SDHA* |  |  |
|  |  |  |  |  |  |  |  |  |  |  | *SDHB* |  |  |
|  |  |  |  |  |  |  |  |  |  |  | *SDHC* |  |  |
|  |  |  |  |  |  |  |  |  |  |  | *SDHD* |  |  |
|  |  |  |  |  |  |  |  |  |  |  | *WRN* |  |  |
|  |  |  |  |  |  |  |  |  |  |  | *CREBBP* |  |  |
|  |  |  |  |  |  |  |  |  |  |  | *DICER1* |  |  |
|  |  |  |  |  |  |  |  |  |  |  | *FOXO1* |  |  |
|  |  |  |  |  |  |  |  |  |  |  | *KRAS* |  |  |
|  |  |  |  |  |  |  |  |  |  |  | *PAX3* |  |  |
|  |  |  |  |  |  |  |  |  |  |  | *PAX7* |  |  |
|  |  |  |  |  |  |  |  |  |  |  | *TNFRSF11A* |  |  |

**Supplementary Table 5 (Online only):** Susceptibility genes for Haematological malignancies (online only) (Adapted from: <http://panelapp.genomicsengland.co.uk>)

| *Haematological* | |
| --- | --- |
| *ACD* | *RMRP* |
| *ANKRD26* | *RPL11* |
| *ATM* | *RPL15* |
| *BLM* | *RPL23* |
| *BRCA1* | *RPL26* |
| *BRCA2* | *RPL31* |
| *BRIP1* | *RPL35A* |
| *CBL* | *RPL36* |
| *CEBPA* | *RPL5* |
| *CTC1* | *RPS10* |
| *DDX41* | *RPS17* |
| *DKC1* | *RPS19* |
| *DNAJC21* | *RPS24* |
| *DOCK8* | *RPS26* |
| *ELANE* | *RPS27* |
| *ERCC4* | *RPS27A* |
| *ERCC6L2* | *RPS28* |
| *ETV6* | *RPS29* |
| *FANCA* | *RPS7* |
| *FANCB* | *RTEL1* |
| *FANCC* | *RUNX1* |
| *FANCD2* | *SAMD9L* |
| *FANCE* | *SBDS* |
| *FANCF* | *SH2D1A* |
| *FANCG* | *SLX4* |
| *FANCI* | *STAT3* |
| *FANCL* | *STN1* |
| *FAS* | *TERC* |
| *GATA1* | *TERT* |
| *GATA2* | *TINF2* |
| *GBA* | *TP53* |
| *HAX1* | *UBE2T* |
| *IKZF1* | *WAS* |
| *ITK* | *WRAP53* |
| *LIG4* | *XRCC2* |
| *MAD2L2* | *FANCM* |
| *MBD4* | *RAD51* |
| *MLH1* | *RAD51C* |
| *MSH2* | *SH2B3* |
| *MSH6* | *STX11* |
| *NAF1* | *STXBP2* |
| *NBN* | *UNC13D* |
| *NF1* | *CSF3R* |
| *NHP2* | *RPL27* |
| *NOP10* | *RPS15* |
| *PALB2* | *TSR2* |
| *PARN* | *UBA2* |
| *PAX5* | *HPLH1* |
| *PMS2* |  |
| *PRF1* |  |
| *PTPN11* |  |
| *RAD21* |  |

**Supplementary Table 6 (online only).** Broader panel of susceptibility genes for adult solid tumours and childhood solid tumours. For Tier 3: panels applied to adult with solid tumours = adult solid tumours, panels applied to children with solid tumours = adult sodlid tumour and childhood solid tumour, panels applied to haematological tumours = adult solid tumours + childhood solid tumours + haematological malignancies; source: <http://panelapp.genomicsengland.co.uk>).

| *Adult Solid Tumours* | | *Childhood Solid Tumours* | |
| --- | --- | --- | --- |
| *APC* | *PTPN11* | *ALK* | *RB1* |
| *ATM* | *RAD51C* | *APC* | *RECQL4* |
| *BAP1* | *RAD51D* | *ATM* | *RET* |
| *BMPR1A* | *RAF1* | *BLM* | *SHOC2* |
| *BRCA1* | *RB1* | *BMPR1A* | *SMAD4* |
| *BRCA2* | *RET* | *BRAF* | *SMARCA4* |
| *BRIP1* | *RTEL1* | *BRCA1* | *SMARCB1* |
| *CBL* | *SDHA* | *BRCA2* | *SOS1* |
| *CDC73* | *SDHAF2* | *BRIP1* | *STK11* |
| *CDH1* | *SDHB* | *BUB1B* | *SUFU* |
| *CDK4* | *SDHC* | *CBL* | *TERT* |
| *CDKN1B* | *SDHD* | *CDKN1C* | *TP53* |
| *CDKN2A* | *SHOC2* | *DDB2* | *TRIP13* |
| *DDB2* | *SMAD4* | *DICER1* | *TSC1* |
| *DICER1* | *SMARCA4* | *DIS3L2* | *TSC2* |
| *EPCAM* | *SMARCB1* | *ELP1* | *VHL* |
| *ERCC2* | *SOS1* | *ERCC1* | *WRN* |
| *ERCC3* | *STK11* | *ERCC2* | *WT1* |
| *ERCC4* | *SUFU* | *ERCC3* | *XPA* |
| *ERCC5* | *TERC* | *ERCC4* | *XPC* |
| *FANCA* | *TERT* | *ERCC5* | *EZH2* |
| *FANCB* | *TMEM127* | *FANCA* | *FANCB* |
| *FANCC* | *TP53* | *FANCC* | *FANCM* |
| *FANCD2* | *TSC1* | *FANCD2* | *GPC3* |
| *FANCE* | *TSC2* | *FANCE* | *NOP10* |
| *FANCF* | *VHL* | *FANCF* | *NSD1* |
| *FANCG* | *WRAP53* | *FANCG* | *PDGFRA* |
| *FANCI* | *WT1* | *FANCI* | *RAD51C* |
| *FANCL* | *XPA* | *FANCL* | *REST* |
| *FH* | *XPC* | *GPR161* | *SLX4* |
| *FLCN* | *ACD* | *HRAS* | *SQSTM1* |
| *HRAS* | *AIP* | *KRAS* | *TRIM37* |
| *KIT* | *BRAF* | *MAP2K1* |  |
| *KRAS* | *CHEK2* | *MAP2K2* |  |
| *MAX* | *CTC1* | *MEN1* |  |
| *MEN1* | *DKC1* | *MLH1* |  |
| *MET* | *ERCC1* | *MSH2* |  |
| *MLH1* | *EXT1* | *MSH6* |  |
| *MSH2* | *EXT2* | *NBN* |  |
| *MSH6* | *LZTR1* | *NF1* |  |
| *MUTYH* | *MAP2K1* | *NF2* |  |
| *NF1* | *MAP2K2* | *NHP2* |  |
| *NF2* | *PARN* | *NRAS* |  |
| *NRAS* | *PPP1CB* | *PALB2* |  |
| *NTHL1* | *RIT1* | *PAX5* |  |
| *PALB2* | *SLX4* | *PHOX2B* |  |
| *PMS2* | *SOS2* | *PMS2* |  |
| *POLD1* | *TINF2* | *PRKAR1A* |  |
| *POLE* | *NOP10* | *PTCH1* |  |
| *POLH* | *PDGFRA* | *PTEN* |  |
| *PTCH1* | *RABL3* | *PTPN11* |  |
| *PTEN* | *SPRED1* | *RAF1* |  |

**Supplementary Table 7 (Online only):** Germline alterations in DPYD reported as of potential pharmacogenomic importance (DPYD deficiency) (adapted from, 100,000 Genomes Project: Validation and Reporting Guidance for DPYD Variants, <https://files.genomicsengland.co.uk/forms/100000-Genomes-Project-Validation-and-Reporting-Guidance-for-DPYD-variants-v1.0-signed.pdf>), last accessed 03.10.2023

| Gene | Nucleotide change | Protein change | rsID |
| --- | --- | --- | --- |
| *DPYD* | c.1905+1G>A |  | rs3918290 |
| *DPYD* | c.1679T>G | p.I560S | rs55886062 |
| *DPYD* | c.2846A>T | p.D949V | rs67376798 |
| *DPYD* | c.1129-5923C>G  c.1236G>A | N/A  p.E412E | rs75017182  rs56038477 |

**Supplementary Table 8 (Online only):** Summary outcomes of all sample sets collected by cancer types (n=4842)*

|  | | All  (n=4842) | Colorectal  (n= 977) | Breast  (n=933) | Urology  (n=680) | Sarcoma  (n=512) | HPB  (n=442) | Lung  (n=327) | Glioma  (n=216) | Blood  (n=185) | Melanoma  (n=177) | Upper GI  (n=154) | H&N  (n=91) | Gynae  (n=73) | Paediatric  (n=53) | CUP  (n=22) | p-value |
| --- | --- | --- | --- | --- | --- | --- | --- | --- | --- | --- | --- | --- | --- | --- | --- | --- | --- |
| Sample sets not sequenced | |  |  |  |  |  |  |  |  |  |  |  |  |  |  |  |  |
| Sample issues | | 913 (54.7) | 126 (51.0) | 158 (49.2) | 149 (62.3) | 61 (38.1) | 125 (72.3) | 73 (60.3) | 31 (38.3) | 46 (44.2) | 36 (56.3) | 34 (60.7) | 42 (75.0) | 16 (64.0) | 14 (82.4) | 2 (40.0) | <0.0001 |
| QC Failure | | 538 (32.2) | 106 (42.9) | 131 (40.8) | 64 (26.8) | 61 (38.1) | 40 (23.1) | 32 (26.4) | 35 (43.2) | 9 (8.7) | 24 (37.5) | 14 (25) | 14 (25.0) | 6 (24.0) | 2 (11.8) | 0 (0) | 0.001 |
| Not eligible | | 63 (3.8) | 1 (0.4) | 5 (1.6) | 6 (2.5) | 4 (2.5) | 2 (1.2) | 5 (4.1) | 2 (2.5) | 34 (32.7) | 2 (3.1) | 1 (1.8) | 0 (0) | 1 (4.0) | 0 (0) | 0 (0) | <0.0001 |
| Unknown | | 155 (9.3) | 14 (5.7) | 27 (8.4) | 20 (8.4) | 34 (21.3) | 6 (3.5) | 11 (9.1) | 13 (16.0) | 15 (14.4) | 2 (3.1) | 7 (12.5) | 0 (0) | 2 (8.0) | 1 (5.9) | 3 (60) | <0.0001 |
| Total* | | 1669 (34.5) | 247 (25.3) | 321 (34.4) | 239 (35.1) | 160 (31.3) | 173 (39.1) | 121 (37.0) | 81 (37.5) | 104 (56.2) | 64 (36.2) | 56 (36.4) | 56 (61.5) | 25 (34.2) | 17 (32.1) | 5 (22.7) | <0.0001 |
| Sample sets sequenced, without result interpreted at last follow up | | | | | | | | | | | | | | | | |  |
| Death | | 320 (17.2) | 55 (11.2) | 17 (4.0) | 40 (16.1) | 33 (24.3) | 36 (31.3) | 41 (28.1) | 34 (54.0) | 9 (18.4) | 24 (44.4) | 17 (27.9) | 5 (22.7) | 4 (18.2) | 0 (0) | 5 (50) | <0.0001 |
| Clinically Excluded | | 1518 (81.5) | 429 (87.4) | 413 (96.0) | 208 (83.9) | 94 (69.1) | 79 (68.7) | 104 (71.2) | 25 (39.7) | 40 (81.6) | 28 (51.9) | 44 (72.1) | 16 (72.7) | 18 (81.8) | 15 (93.8) | 5 (50) | <0.0001 |
| Awaited / Unknown | | 25 (1.3) | 7 (1.4) | 0 (0) | 0 (0) | 9 (6.6) | 0 (0) | 1 (0.7) | 4 (6.3) | 0 (0) | 2 (3.7) | 0 (0) | 1 (4.5) | 0 (0) | 1 (6.3) | 0 (0) | 0.001 |
| Total* | | 1863 (38.5) | 491 (50.3) | 430 (46.1) | 248 (36.5) | 136 (26.6) | 115 (26.0) | 146 (44.6) | 63 (29.2) | 49 (26.5) | 54 (30.5) | 61 (39.6) | 22 (24.2) | 22 (30.1) | 16 (30.2) | 10 (45.5) | <0.0001 |
| Sample sets sequenced, with results interpreted at the last follow up | | | | | | | | | | | | | | | | |  |
| No Actionable Domain 1 Variant | | 336 (25.6) | 33 (13.8) | 73 (40.1) | 96 (49.7) | 24 (13.3) | 51 (33.1) | 18 (30.0) | 4 (5.6) | 0 (0) | 9 (15.3) | 12 (32.4) | 6 (46.2) | 3 (11.5) | 2 (10.0) | 5 (71.4) | <0.0001 |
| Listed for GTAB | | 969 (74.0) | 205 (85.8) | 108 (59.3) | 96 (49.7) | 192 (86.7) | 102 (66.2) | 42 (70.0) | 68 (94.4) | 32 (100) | 50 (84.7) | 25 (67.6) | 7 (53.8) | 23 (88.5) | 17 (85.0) | 2 (28.6) | <0.0001 |
| Report without GTAB | | 5 (0.4) | 1 (0.4) | 1 (0.5) | 1 (0.5) | 0 (0) | 1 (0.6) | 0 (0) | 0 (0) | 0 (0) | 0 (0) | 0 (0) | 0 (0) | 0 (0) | 1 (5.0) | 0 (0) | ns |
| Total* | 1310 (27.1) | 239 (24.5) | 182 (19.5) | 193 (28.4) | 216 (42.2) | 154 (34.8) | 60 (18.3) | 72 (33.3) | 32 (17.3) | 59 (33.3) | 37 (24.0) | 13 (14.3) | 26 (35.6) | 20 (37.7) | 7 (31.8) | <0.0001 |  |
|  |  |  |  |  |  |  |  |  |  |  |  |  |  |  |  |  |  |

*9 sample sets had no cancer site recorded (9/4851; 0.18%); Count (percentage of category); *Count (Percentage of column total); HPB= hepatobiliary; Blood= haematological; GI= gastrointestinal tract; H&N= head and neck; Gynae= Gynaecological; CUP= Cancer of Unknown Primary. 5 Samples had reports issued without GTAB discussion

**Supplementary Table 9 (Online only):** A summary of recommended licensed/unlicensed treatment by genetic aberrations (n=144).

| **Recommended therapeutics**  **(n=144)** | **Number (%)** | **Genetic Aberrations**  **Number (%)** | | | | | | |
| --- | --- | --- | --- | --- | --- | --- | --- | --- |
| Checkpoint Inhibitors | 97 (67.4) | High TMB  96 (99.0) | *MLH1*  1 (1.0) |  |  |  |  |  |
| *PI3K/Akt/mTOR* Inhibitors | 21 (14.6) | *PIK3CA*  10 (47.6) | *MTOR*  5 (23.8) | *TSC1*  2 (9.5) | *PTEN*  1 (4.8) | *AKT1*  1 (4.8) | *STK11*  1 (4.8) | *TSC2*  1 (4.8) |
| *BRAF* and/or *MEK* Inhibitors | 12 (8.3) | *BRAF*  7 (58.3) | *NRAS*  3 (25.0) | *MAP2K4*  2 (16.7) |  |  |  |  |
| PARP Inhibitors | 8 (5.5) | *ATM*  3 (37.5) | *PALB2*  3 (37.5) | *BRCA1*  1 (12.5) | *BRCA2*  1 (12.5) |  |  |  |
| *KRAS* Targeted Therapy | 2 (1.4) | *KRAS*  2 (100.0) |  |  |  |  |  |  |
| *IDH1* Targeted Therapy | 1 (0.7) | *IDH1*  1 (100.0) |  |  |  |  |  |  |
| Imatinib | 1 (0.7) | *ATR, KIT*  1 (100.0) |  |  |  |  |  |  |
| *MAP2K4* Targeted Therapy | 1 (0.7) | *MAP2K4*  1 (100.0) |  |  |  |  |  |  |

**Supplementary Table 10 (Online only):** Representing genes of germline variants identified by cancer type (n=82)

| Gene | Total  (n= 82) | Colorectal  (n= 18) | Breast  (n=28) | Urology  (n= 9 ) | Sarcoma  (n=11) | HPB  (n=6) | Lung  (n=1) | Glioma  (n=3) | Blood  (n=2) | Melanoma  (n=1) | Upper GI  (n=0) | H&N  (n=0) | Gynae  (n=0) | Paediatric  (n=3) | CUP  (n=0) |
| --- | --- | --- | --- | --- | --- | --- | --- | --- | --- | --- | --- | --- | --- | --- | --- |
| *BRCA2* | 21 (25.6) | 4 (22.2) | 14 (50.0) | 0 (0) | 3 (27.3) | 0 (0) | 0 (0) | 0 (0) | 0 (0) | 0 (0) | 0 (0) | 0 (0) | 0 (0) | 0 (0) | 0 (0) |
| *MSH6* | 11 (13.4) | 5 (27.8) | 1 (3.6) | 1 (11.1) | 1 (9.1) | 3 (50.0) | 0 (0) | 0 (0) | 0 (0) | 0 (0) | 0 (0) | 0 (0) | 0 (0) | 0 (0) | 0 (0) |
| *ATM* | 8 (9.8) | 1 (5.6) | 2 (7.1) | 2 (22.2) | 1 (9.1) | 1 (16.7) | 1 (100.0) | 0 (0) | 0 (0) | 0 (0) | 0 (0) | 0 (0) | 0 (0) | 0 (0) | 0 (0) |
| *BRIP1* | 7 (8.5) | 1 (5.6) | 3 (10.7) | 1 (11.1) | 1 (9.1) | 0 (0) | 0 (0) | 0 (0) | 1 (50.0) | 0 (0) | 0 (0) | 0 (0) | 0 (0) | 0 (0) | 0 (0) |
| *PALB2* | 5 (6.1) | 0 (0) | 3 (10.7) | 1 (11.1) | 1 (9.1) | 0 (0) | 0 (0) | 0 (0) | 0 (0) | 0 (0) | 0 (0) | 0 (0) | 0 (0) | 0 (0) | 0 (0) |
| *TP53* | 4 (4.9) | 0 (0) | 1 (3.6) | 0 (0) | 3 (27.3) | 0 (0) | 0 (0) | 0 (0) | 0 (0) | 0 (0) | 0 (0) | 0 (0) | 0 (0) | 0 (0) | 0 (0) |
| *BRCA1* | 4 (4.9) | 0 (0) | 2 (7.1) | 1 (11.1) | 1 (9.1) | 0 (0) | 0 (0) | 0 (0) | 0 (0) | 0 (0) | 0 (0) | 0 (0) | 0 (0) | 0 (0) | 0 (0) |
| *MLH1* | 3 (3.7) | 2 (11.1) | 0 (0) | 1 (11.1) | 0 (0) | 0 (0) | 0 (0) | 0 (0) | 0 (0) | 0 (0) | 0 (0) | 0 (0) | 0 (0) | 0 (0) | 0 (0) |
| *PMS2* | 3 (3.7) | 1 (5.6) | 1 (3.6) | 1 (11.1) | 0 (0) | 0 (0) | 0 (0) | 0 (0) | 0 (0) | 0 (0) | 0 (0) | 0 (0) | 0 (0) | 0 (0) | 0 (0) |
| *RAD51D* | 2 (2.4) | 0 (0) | 0 (0) | 0 (0) | 0 (0) | 0 (0) | 0 (0) | 1 (33.3) | 0 (0) | 0 (0) | 0 (0) | 0 (0) | 0 (0) | 1 (33.3) | 0 (0) |
| *FLCN* | 2 (2.4) | 1 (5.6) | 0 (0) | 1 (11.1) | 0 (0) | 0 (0) | 0 (0) | 0 (0) | 0 (0) | 0 (0) | 0 (0) | 0 (0) | 0 (0) | 0 (0) | 0 (0) |
| *BAP1* | 1 (1.2) | 0 (0) | 0 (0) | 0 (0) | 0 (0) | 0 (0) | 0 (0) | 0 (0) | 0 (0) | 1 (100.0) | 0 (0) | 0 (0) | 0 (0) | 0 (0) | 0 (0) |
| *SDHA* | 1 (1.2) | 1 (5.6) | 0 (0) | 0 (0) | 0 (0) | 0 (0) | 0 (0) | 0 (0) | 0 (0) | 0 (0) | 0 (0) | 0 (0) | 0 (0) | 0 (0) | 0 (0) |
| *POLD1* | 1 (1.2) | 1 (5.6) | 0 (0) | 0 (0) | 0 (0) | 0 (0) | 0 (0) | 0 (0) | 0 (0) | 0 (0) | 0 (0) | 0 (0) | 0 (0) | 0 (0) | 0 (0) |
| *NF1* | 1 (1.2) | 0 (0) | 0 (0) | 0 (0) | 0 (0) | 1 (16.7) | 0 (0) | 0 (0) | 0 (0) | 0 (0) | 0 (0) | 0 (0) | 0 (0) | 0 (0) | 0 (0) |
| *SUFU* | 1 (1.2) | 0 (0) | 0 (0) | 0 (0) | 0 (0) | 0 (0) | 0 (0) | 0 (0) | 0 (0) | 0 (0) | 0 (0) | 0 (0) | 0 (0) | 1 (33.3) | 0 (0) |
| *APC* | 1 (1.2) | 0 (0) | 0 (0) | 0 (0) | 0 (0) | 1 (16.7) | 0 (0) | 0 (0) | 0 (0) | 0 (0) | 0 (0) | 0 (0) | 0 (0) | 0 (0) | 0 (0) |
| *RET* | 1 (1.2) | 0 (0) | 1 (3.6) | 0 (0) | 0 (0) | 0 (0) | 0 (0) | 0 (0) | 0 (0) | 0 (0) | 0 (0) | 0 (0) | 0 (0) | 0 (0) | 0 (0) |
| *POLE* | 1 (1.2) | 0 (0) | 0 (0) | 0 (0) | 0 (0) | 0 (0) | 0 (0) | 1 (33.3) | 0 (0) | 0 (0) | 0 (0) | 0 (0) | 0 (0) | 0 (0) | 0 (0) |
| *MSH2* | 1 (1.2) | 1 (5.6) | 0 (0) | 0 (0) | 0 (0) | 0 (0) | 0 (0) | 0 (0) | 0 (0) | 0 (0) | 0 (0) | 0 (0) | 0 (0) | 0 (0) | 0 (0) |
| *NF2* | 1 (1.2) | 0 (0) | 0 (0) | 0 (0) | 0 (0) | 0 (0) | 0 (0) | 0 (0) | 0 (0) | 0 (0) | 0 (0) | 0 (0) | 0 (0) | 1 (33.3) | 0 (0) |
| *TSC2* | 1 (1.2) | 0 (0) | 0 (0) | 0 (0) | 0 (0) | 0 (0) | 0 (0) | 0 (0) | 1 (50.0) | 0 (0) | 0 (0) | 0 (0) | 0 (0) | 0 (0) | 0 (0) |
| *RAD51C* | 1 (1.2) | 0 (0) | 0 (0) | 0 (0) | 0 (0) | 0 (0) | 0 (0) | 1 (33.3) | 0 (0) | 0 (0) | 0 (0) | 0 (0) | 0 (0) | 0 (0) | 0 (0) |

Count (%). One patient had mutation in both ATM and PMS2. 4 patients had no identified germline variant but were referred to clinical genetics based on clinical history.

Supplementary Table 10: A summary of recommended clinical trials (n=348)

**Supplementary Table 11 (Online only):** A summary of recommended clinical trials (n=348).

| **Clinical Trial** | **Phase** | **Population**  ***Genetic Selection*** | **Targeted therapy** | **Recommendations (%)** |
| --- | --- | --- | --- | --- |
| NCT03096054 | I | Advanced cancer  *Enriched for TP53 mutation* | LY3143921  (CDC7i) | 76 (21.8) |
| NCT02407509 | I | Solid tumour or multiple myeloma  *BRAF, KRAS, NRAS mutant* | VS-6766  (RAF/MEKi) | 50 (14.4) |
| NCT03188965 | I | Advanced Solid Tumour or Lymphoma  *Deoxyribonucleic Acid Damage Repair (e.g. ATM mutant)* | Elimusertib  (ATRi) | 41 (11.8) |
| NCT03673787 | I/II | Advanced solid tumours (or potentially resectable glioblastoma).  *Hyperactivation of PI3K pathway (cohort B1)* | Ipatasertib  (Akti) | 40 (11.5) |
| NCT03684811 | I/II | Advanced solid tumours and glioma  *IDH1 mutant* | Olutasidenib  (IDH1i) | 24 (6.9) |
| NCT02157792 | I | Advanced solid tumours  *TP53 mutation and/or loss of ATM expression (Cohort C1)* | Berzosertib  (ATRi) | 18 (5.2) |
| NCT02797964 | I/II | Advanced solid tumours  *Predicted sensitivity to ChK1 INHIBITION* | SRA737  (Chk1i) | 17 (4.9) |
| NCT02599324 | I/II | Advanced Gastrointestinal and Genitourinary tumours  *KRAS wt BRAF wt* | Ibrutinib | 9 (2.6) |
| NCT02664935 | II | Advanced Lung Cancer (*Umbrella Trial )*  *FGFR2/3 mut, TSC1/2 mut, STK11 mut, CDKN3A loss, CDK4 amp, CCND1 amp, KRAS mut, MET amp, ROS1 fusion, MET exon 14 skipping, NF1 mut, NRAS mut, PIK3CA mut, PIK3CA amp, PTEN loss, EGFR mut, RET rearrangement* | Platform Trial | 9 (2.6) |
| NCT03056755 | II | Hormone Receptor positive, HER2 negative  *PIK3CA mutant* | Alpelisib  (PI3Ki) | 8 (2.3) |
| NCT03006172 | I | Locally advanced/metastatic solid tumours; Locally advanced/metastatic breast cancer  *PIK3CA mutant* | Inavolisib  (PI3Ki) | 7 (2.0) |
| NCT03337724 | III | Locally advanced or metastatic Triple-Negative Breast Cancer  Locally advanced or metastatic HR+, HER2 -Breast Cancer  *PIK3CA/Akt1/PTEN altered* | Ipatasertib  (Akti) | 7 (2.0) |
| NCT02052778 | I/II | Advanced solid tumours (intrahepatic cholangiocarcinoma Phase II)  *FGF/FGFR aberrations* | Futibatinib  (FGFRi) | 5 (1.4) |
| NCT02797977 | I/II | Advanced Cancer  *Predicted sensitivity to ChK1 Inhibition* | SRA737  (Chk1i) | 5 (1.4) |
| NCT01884285 | I | Advanced castrate-resistant prostate, squamous lung, triple negative breast cancer  *PTEN deficient/mutant, PIK3CB mutant/amplified* | AZD8186  (PI3Kβ/δi) | 4 (1.1) |
| NCT02264678 | I | Advanced Cancer (*Modular design)*  *ATM deficient/proficient gastric, NSCLC, breast, ovarian, head and neck squamous* | Ceralasertib  (ATRi) | 4 (1.1) |
| NCT02338622 | I | Advanced solid tumours  *Germline BRCA1/2 mut (Cohort 1), Sporadic cancers with homologous recombination defects or mutations resulting in hyperactivated PI3K/Akt pathway (Cohort 2)* | Olaparib (PARPi) and Capivasertib (Akti) | 4 (1.1) |
| NCT02291289 | II | Metastatic Colorectal Cancer  *BRAF mutant, PIK3CA mutant, PTEN loss, KRAS mutant, NRAS mutant, TP53 mutant, all wild-type* | Platform Trial | 3 (0.9) |
| NCT03473743 | I/II | Metastatic or Locally Advanced Urothelial Cancer  *FGFR3/2 Alteration* | Erdafitinib  (FGFRi) | 3 (0.9) |
| NCT01677741 | I/II | Paediatric Advanced Solid Tumours  *BRAF V600 Mutant* | Dabrafenib  `(BRAFi) | 2 (0.6) |
| NCT01953926 | II | Advanced solid tumours  *HER2 or EGFR exon 18 mutations* | Neratinib (TKI) | 2 (0.6) |
| NCT02872714 | II | Metastatic or surgically unresectable urothelial carcinoma  *FGF/FGFR alteration* | Pemigatinib  (FGFRi) | 2 (0.6) |
| NCT02183883 | II | Advanced NSCLC  *EGFR or HER2 mutation* | Afatinib | 1 (0.3) |
| NCT02389842 | I | Advanced solid cancers. Advanced Breast cancer  *PIK3CA mutant* | Taselisib /Pictilisib + Palbociclib  (PI3Ki, CDK4/6i) | 1 (0.3) |
| NCT02546661 | I | Muscle Invasive Bladder Cancer  *FGFR mut, ATM mut, BRCA1/2 mutant, HRR gene mut, RICTOR mut, TSC1/2 mut* | Platform Trial | 1 (0.3) |
| NCT02631447 | II | Metastatic Melanoma  *BRAF mut* | Encorafenib and Binimetinib  (BRAFi, MEKi) | 1 (0.3) |
| NCT02983045 | I/II | Advanced or metastatic solid tumour  (Including melanoma with known BRAF status) | NKTR-214 | 1 (0.3) |
| NCT03377361 | I/II | Metastatic colorectal cancer  (Excluding BRAF mutant) | Nivolumab + Trametinib +/- ipilimumab | 1 (0.3) |
| NCT03384940 | II | Advanced colorectal cancer  *HER2 positive* | Trastuzumab Deruxtecan | 1 (0.3) |
| NCT03827044 | III | Stage III colon cancer  *MSI-High or POLE mutant* | Avelumab + 5FU | 1 (0.3) |

**Supplementary Table 12 (Online only):** Summary of clinical follow up of all recommendations by cancer type (n=718)

|  | **All**  **(n=718)** | **Colorectal**  **(n=163)** | **Breast**  **(n=109)** | **Urology**  **(n=71)** | **Sarcoma**  **(n=68)** | **HPB**  **(n=80)** | **Lung**  **(n=56)** | **Glioma**  **(n=60)** | **Blood**  **(n=14)** | **Melanoma**  **(n=49)** | **Upper GI**  **(n=15)** | **H&N**  **(n=5)** | **Gynae**  **(n=16)** | **Paediatric**  **(n=11)** | **CUP**  **(n=1)** |  |
| --- | --- | --- | --- | --- | --- | --- | --- | --- | --- | --- | --- | --- | --- | --- | --- | --- |
| **Recommendations followed** | |  |  |  |  |  |  |  |  |  |  |  |  |  |  |  |
| Referral to clinical genetics | 79 (54.5) | 19 (61.3) | 27 (65.9) | 6 (54.5) | 11 (40.7) | 7 (77.8) | 1 (12.5) | 2 (40.0) | 1 (33.3) | 1 (100.0) | 0 (0) | 0 (0) | 0 (0) | 4 (100.0) | 0 | <0.0001 |
| Patient informed (DPD) | 40 (27.6) | 7 (22.6) | 9 (22.0) | 5 (45.5) | 9 (33.3) | 0 (0) | 6 (75.0) | 0 (0) | 2 (66.7) | 0 (0) | 0 (0) | 1 (100.0) | 1 (33.3) | 0 (0) | 0 | 0.0056 |
| Referred to Trial | 24 (16.6) | 5 (16.1) | 3 (7.3) | 0 (0) | 7 (25.9) | 2 (22.2) | 1 (12.5) | 3 (60.0) | 0 (0) | 0 (0) | 1 (100.0) | 0 (0) | 2 (66.7) | 0 (0) | 0 | ns |
| Compassionate Treatment | 2 (1.4) | 0 (0) | 2 (4.9) | 0 (0) | 0 (0) | 0 (0) | 0 (0) | 0 (0) | 0 (0) | 0 (0) | 0 (0) | 0 (0) | 0 (0) | 0 (0) | 0 | ns |
| **Total recommendations followed** | **145 (20.2)** | **31 (19.0)** | **41 (37.6)** | **11 (15.5)** | **27 (39.7)** | **9 (11.3)** | **8 (14.3)** | **5 (8.3)** | **3 (21.4)** | **1 (2.0)** | **1 (6.7)** | **1 (20.0)** | **3 (18.8)** | **4 (36.4)** |  |  |
|  |  |  |  |  |  |  |  |  |  |  |  |  |  |  |  |  |
| **Recommendation not followed** | |  |  |  |  |  |  |  |  |  |  |  |  |  |  |  |
| RIP | 82 (16.1) | 19 (15.6) | 6 (9.8) | 7 (15.6) | 0 (0) | 17 (24.3) | 16 (42.1) | 10 (18.5) | 0 (0) | 4 (10.5) | 2 (15.4) | 0 (0) | 0 (0) | 1 (20.0) | 0 (0) | <0.0001 |
| Patient not fit / suitable | 46 (9.1) | 13 (10.7) | 2 (3.3) | 6 (13.3) | 4 (10.0) | 6 (8.6) | 1 (2.6) | 7 (13.0) | 0 (0) | 4 (10.5) | 2 (15.4) | 0 (0) | 1 (9.1) | 0 (0) | 0 (0) | ns |
| No disease | 201 (39.6) | 58 (47.5) | 26 (42.6) | 16 (35.6) | 27 (67.5) | 23 (32.9) | 10 (26.3) | 14 (25.9) | 0 (0) | 12 (31.6) | 4 (30.8) | 2 (50.0) | 7 (63.6) | 1 (20.0) | 1 (100.0) | <0.05 |
| No change to treatment | 169 (33.3) | 31 (25.4) | 27 (44.3) | 16 (35.6) | 9 (22.5) | 21 (30.0) | 9 (23.7) | 22 (40.7) | 5 (83.3) | 18 (47.4) | 4 (30.8) | 2 (50.0) | 2 (18.2) | 3 (60.0) | 0 (0) | ns |
| Patient Declined | 5 (1.0) | 1 (0.8) | 0 (0) | 0 (0) | 0 (0) | 3 (4.3) | 0 (0) | 0 (0) | 0 (0) | 0 (0) | 0 (0) | 0 (0) | 1 (9.1) | 0 (0) | 0 (0) | ns |
| Not relevant | 3 (0.6) | 0 (0) | 0 (0) | 0 (0) | 0 (0) | 0 (0) | 1 (0) | 1 (1.9) | 1 (16.7) | 0 (0) | 0 (0) | 0 (0) | 0 (0) | 0 (0) | 0 (0) | <0.05 |
| Different recommendation followed | 1 (0.2) | 0 (0) | 0 (0) | 0 (0) | 0 (0) | 0 (0) | 1 (1.8) | 0 (0) | 0 (0) | 0 (0) | 0 (0) | 0 (0) | 0 (0) | 0 (0) | 0 (0) | ns |
| Unable to contact | 1 (0.2) | 0 (0) | 0 (0) | 0 (0) | 0 (0) | 0 (0) | 0 (0) | 0 (0) | 0 (0) | 0 (0) | 1 (7.7) | (0) | 0 (0) | 0 (0) | 0 (0) | ns |
| **Total recommendations not followed** | **508 (70.8)** | **122 (74.8)** | **61 (56.0)** | **45 (63.4)** | **40 (58.8)** | **70 (87.5)** | **38 (67.9)** | **54 (90.0)** | **6 (42.8)** | **38 (77.6)** | **13 (86.7)** | **4 (80.0)** | **11 (68.8)** | **5 (45.5)** | **1 (100.0)** | **<0.0001** |
|  |  |  |  |  |  |  |  |  |  |  |  |  |  |  |  |  |
| Incomplete data | 65 (9.1) | 10 (6.1) | 7 (6.4) | 15 (21.1) | 1 (1.5) | 1 (1.3) | 10 (17.9) | 1 (1.7) | 5 (35.7) | 10 (20.4) | 1 (6.7) | 0 (0) | 2 (12.5) | 2 (18.2) | 0 (0) | **<0.0001** |
| **Total recommendations** | **718** | **163** | **109** | **71** | **68** | **80** | **56** | **60** | **14** | **49** | **15** | **5** | **16** | **11** | **1** |  |

Count (percentage of category); *Count (Percentage of column total); HPB= hepatobiliary; Blood= haematological; GI= gastrointestinal tract; H&N= head and neck; Gynae= Gynaecological; CUP= Cancer of Unknown Primary; DPYD= dihydropyrimidine dehydrogenase polymorphism; TMB= Tumour mutational burden

**1.3 Supplementary Figures**

**Supplementary Figure 1:** Schema for interpretation of WGS data through pre-GTAB filtering and GTAB. Pre-GTAB Filtering (blue decision points) were designed to identify patients that would not benefit from GTAB data review. All germline findings were discussed at GTAB. Alive patients, on active cancer treatment or high risk of relapse, with reported variants in Domain 1 genes were discussed at GTAB. Variants were discussed at GTAB, with only potentially clinically actionable (likely pathogenic) variants being reported back to the clinical team with recommended actions as outlined.


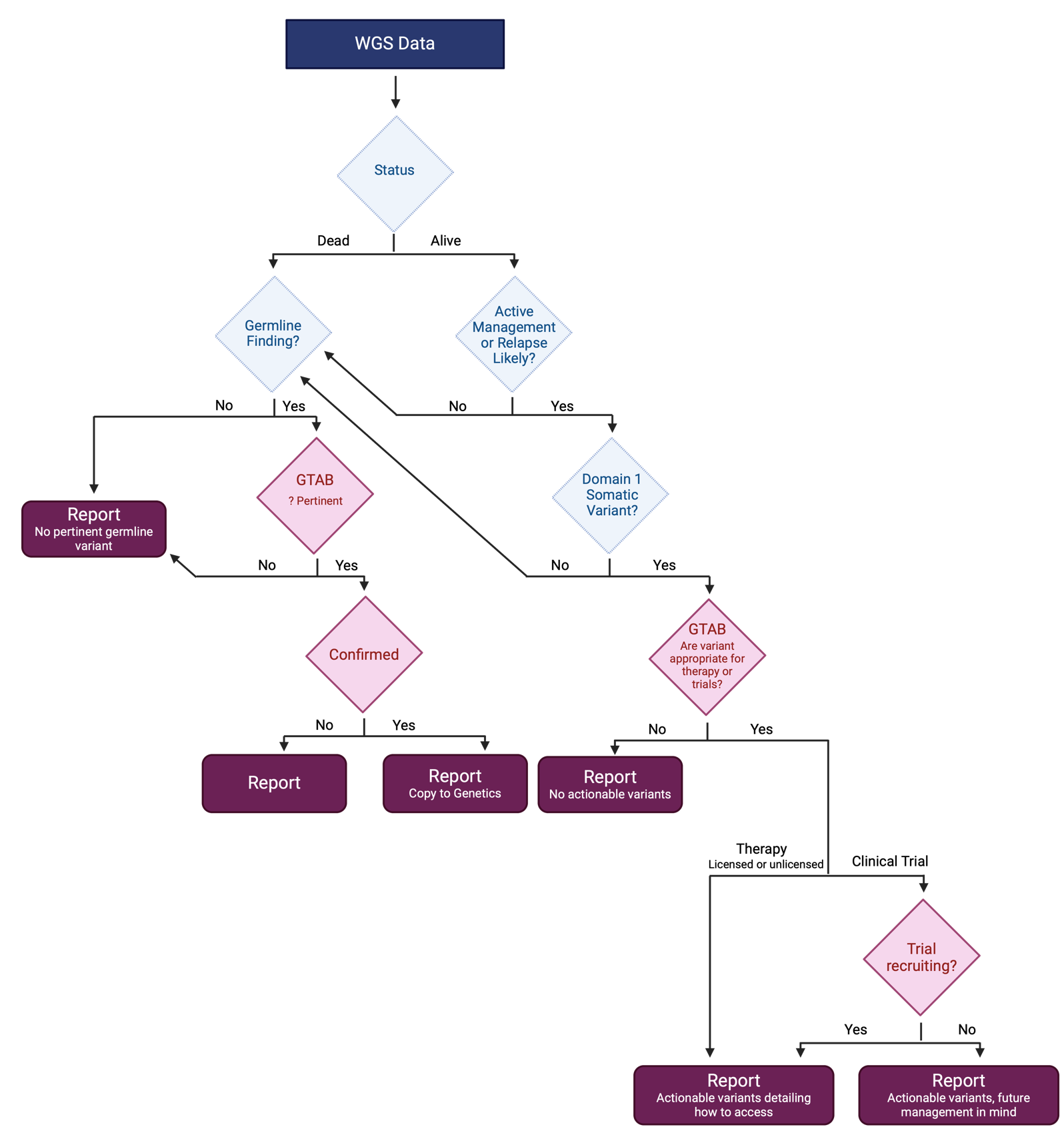


**Supplementary Figure 2:** A flow chart summarising the attrition from initial paired samples to the number of participants with sequenced data.

**Paired samples (germline and somatic)**

(n=4851)

**No linked sequencing data**

Samples not sequenced (n=1669; 34%)

Cancer type not recorded (n=9; 0.2%)

**Remove duplicated patient samples**

(n=106; 2%)

**Paired samples sequenced**

(n=3173; 65%)

**Participants with sequencing data**

(n=3067)

**Supplementary Figure 3:** The number of paired germline and somatic samples included over time within the West Midlands GMC (n=4851)

**Supplementary Figure 4:** Representing genes of variants identified that led to therapeutic recommendations (clinical trial and licensed/unlicensed treatments).
